# *In vivo* anti‐V‐ATPase antibody treatment delays ovarian tumor growth by increasing antitumor immune responses

**DOI:** 10.1002/1878-0261.12782

**Published:** 2020-09-10

**Authors:** Arpita Kulshrestha, Gajendra K. Katara, Safaa A. Ibrahim, Valerie E. Riehl, Sylvia Schneiderman, Mahmood Bilal, Alexandria N. Young, Shayna Levine, Sara Fleetwood, James Dolan, Alice Gilman‐Sachs, Kenneth D. Beaman

**Affiliations:** ^1^ Department of Microbiology and Immunology Center for Cancer cell biology, Immunology and infection Rosalind Franklin University of Medicine and Science North Chicago IL USA; ^2^ Department of Microbiology and Immunology Faculty of Pharmacy Cairo University Giza Egypt; ^3^ Center for Biomolecular Sciences College of Pharmacy University of Illinois at Chicago IL USA; ^4^ Chicago Medical School Rosalind Franklin University of Medicine and Science North Chicago IL USA; ^5^ Department of Obstetrics & Gynecology Advocate Lutheran General Hospital Park Ridge IL USA

**Keywords:** a2 isoform, monoclonal antibody, ovarian cancer, vacuolar‐ATPase

## Abstract

Tumor acidity is the key metabolic feature promoting cancer progression and is modulated by pH regulators on a cancer cell's surface that pump out excess protons/lactic acid for cancer cell survival. Neutralizing tumor acidity improves the therapeutic efficacy of current treatments including immunotherapies. Vacuolar‐ATPase (V‐ATPase) proton pumps encompass unique plasma membrane‐associated subunit isoforms, making this molecule an important target for anticancer therapy. Here, we examined the *in vivo* therapeutic efficacy of an antibody (a2v‐mAB) targeting specific V‐ATPase‐‘V0a2’ surface isoform in controlling ovarian tumor growth. *In vitro* a2v‐mAb treatment inhibited the proton pump activity in ovarian cancer (OVCA) cells. *In vivo* intraperitoneal a2v‐mAb treatment drastically delayed ovarian tumor growth with no measurable *in vivo* toxicity in a transplant tumor model. To explore the possible mechanism causing delayed tumor growth, histochemical analysis of the a2v‐mAb‐treated tumor tissues displayed high immune cell infiltration (M1‐macrophages, neutrophils, CD103^+^ cells, and NK cells) and an enhanced antitumor response (iNOS, IFN‐y, IL‐1α) compared to control. There was marked decrease in CA‐125‐positive cancer cells and an enhanced active caspase‐3 expression in a2v‐mAb‐treated tumors. RNA‐seq analysis of a2v‐mAb tumor tissues further revealed upregulation of apoptosis‐related and toll‐like receptor pathway‐related genes. Indirect coculture of a2v‐mAb‐treated OVCA cells with human PBMCs in an unbuffered medium led to an enhanced gene expression of antitumor molecules IFN‐y, IL‐17, and IL‐12‐A in PBMCs, further validating the *in vivo* antitumor responses. In conclusion, V‐ATPase inhibition using a monoclonal antibody directed against the V0a2 isoform increases antitumor immune responses and could therefore constitute an effective treatment strategy in OVCA.

## Graphical abstract

Tumor acidity is the key metabolic feature promoting cancer progression by eliciting immune‐suppression. V‐ATPases on a cancer cell's surface pump out excess protons and acidify the tumor microenvironment (TME). *In vivo* treatment of ovarian tumors using a monoclonal antibody (a2v‐mAb) directed against V‐ATPase‐V0a2 delays tumor growth by enhancing antitumor immune responses, making it an effective treatment strategy in ovarian cancer.

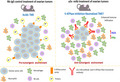

**Abbreviations**
OVCA: ovarian cancer
TME: tumor microenvironment
V‐ATPase: Vacuolar‐ATPase

## 1 Introduction

Ovarian cancer (OVCA), the most lethal gynecological malignancy, accounts for an estimated 295 000 new cases and 184 000 deaths worldwide annually [1]. The high mortality rate in OVCA due to delayed diagnosis and chemoresistance in relapse patients is currently the most pressing concern. Effective treatments for OVCA patients to treat disease relapse are requisite to improve the patient survival rates[2].

Current OVCA treatment options include standard chemotherapy, radiotherapy, as well as immune checkpoint blockade (ICB) therapy [3, 4]. Several factors in the ovarian tumor microenvironment (TME) impair antitumor cell function that makes ovarian TME immunosuppressive and leads to cancer progression. Knowledge of tumor‐associated antigens and the surrounding TME is therefore essential to explore ways to increase the tumor immunogenicity and improve responses to treatment [5].

The key difference between tumors and the surrounding normal tissue is the nutritional and metabolic environment. These physiological factors in the TME play a fundamental role in fabricating an immune‐suppressive environment. The tumor acidity is emerging as a key modulator of cancer‐related immunosuppression that facilitates disease spread [6]. Neutralizing the tumor pH inhibits cancer growth [7] and improves response to immunotherapies such as anti‐PD‐1 and anti‐CTLA‐4 [8]. This acid extrusion into the TME requires a specific repertoire of pH regulatory molecules on cancer cell surface [9, 10, 11]. Targeting tumor pH regulators is therefore an attractive avenue for anticancer therapies.

One of the primary pH regulators is the proton pump vacuolar H+‐ ATPases (V‐ATPases) [12] that are multisubunit, ATP‐dependent proton pumps functioning in a vast array of normal cellular processes such as protein processing/degradation, membrane trafficking as well as special physiological functions such as bone resorption, urinary acidification [13]. The different V‐ATPase subunit isoforms are expressed in cell or organelle‐specific manner [14]. In tumors, the V‐ATPases are overexpressed on a wide array of cancer cells where they contribute to tumor acidification [15]. Since most of the V‐ATPase subunits and isoforms are involved in key physiology in normal cells, scanning for the cancer‐specific V‐ATPase subunit isoforms is critical for avoiding toxicity issues. Previous studies established that a specific ‘a2’ subunit isoform of V‐ATPase membrane‐bound V0 domain (V‐ATPase‐V0a2) is distinctly expressed on malignant ovarian cell surface and absent on normal cells [16] and also contributes to cancer immune modulation [16, 17]. V‐ATPase‐V0a2 gene knockdown restores cisplatin activity in drug‐resistant OVCA cells [18]. *In vitro* V‐ATPase‐V0a2 inhibition by monoclonal antibody impedes cancer cell migration and MMPs activity in OVCA cells [16]. In breast cancer, *in vivo* tumor growth is delayed by V‐ATPase‐V0a2 knockdown in cancer cells due to an altered TME [19].

The present study investigated the therapeutic efficacy of an antagonist monoclonal antibody to a tumor‐specific isoform of V‐ATPase (V‐ATPase‐V0a2) as a strategy to treat OVCA. This anti‐V‐ATPase‐V0a2 antibody (a2v‐mAb) can effectively recognize both mouse and human V‐ATPases. In the current study, human ovarian tumors were established in the athymic nude mice and treated with a2v‐mAb and monitored for tumor progression. *In vitro*, a2v‐mAb treatment inhibited proton pump activity in OVCA cells. *In vivo* a2v‐mAb treatment resulted in delayed tumor growth, with no *in vivo* toxicity in nude mice. Tumors from a2v‐mAb‐treated mice revealed high immune cell infiltration, an elevated antitumor responses, and a marked decrease in cancer cell numbers. RNA sequencing analysis confirmed high expression of antitumor cytokines and toll‐like receptor (TLR) pathway in a2v‐mAb TME. This study establishes the utility of antibody‐based V‐ATPase inhibition in controlling OVCA growth by changing the tumor immune landscape by altering the tumor acidity.

## 2 Material and methods

### 2.1 Cell Lines and cell culture

The human ovarian carcinoma cell lines A2780 and SKOV‐3 were used in this study. A2780 cell line was procured from Sigma‐Aldrich, St Louis, MO, USA, and cultured in RPMI 1640 medium (Invitrogen, Carlsbad, CA, USA). SKOV‐3 cell line was procured from ATCC, Manassas, VA, USA, and cultured in McCoy's 5a medium (ATCC). All media were supplemented with 10% (v/v) heat‐inactivated fetal bovine serum (Biowest LLC, Riverside, MO, USA), 100 U·mL^−1^ penicillin, and 100 U·mL^−1^ streptomycin (Sigma‐Aldrich). For routine culture, cells were grown until reaching approximately 80% confluency and then plated for experiments.

### 2.2 Generation of monoclonal antibody to V‐ATPase‐V0a2

The murine hybridoma cell line was used to generate a2v‐mAb as described previously [20, 21]. This hybridoma was produced using a synthetic peptide that represents amino acids 488–510 of the membrane‐bound portion of V‐ATPase‐V0a2 (previously referred to as regeneration and tolerance factor; RTF). For ascites production, hybridoma cell line was grown as described previously [21] in RPMI medium supplemented with 10% FCS. Monoclonal antibody (a2v‐mAb) was purified using proteinG column (Covance Inc., Denver, PA, USA). The specificity of the antibody was assessed using two different isotype‐control antibodies [21]. Functionally, this antibody has been shown to inhibit the migration and suppress matrix metalloproteinase activity (MMP‐9 & MMP‐2) activity OVCA cells [16]. Further, a2v‐mAb treatment increases the cellular cytosolic pH of OVCA cells [18].

### 2.3 *In vivo* ovarian tumor generation and antibody treatment

Female athymic nude mice, 4 weeks old, were purchased from Charles River (New York, NY, USA). Animal care and all animal experiments were in accordance with the guidelines of Rosalind Franklin University IACUC Committee. The injection materials (cell lines and antibodies) were sterile and free of any LPS or mycobacterial contamination. For the ovarian tumor experiments, on day 0, animals were injected subcutaneously (s.c.) in the upper flank region with 200 µL (1 : 1 slurry) of Matrigel mix and PBS containing 0.3 × 10^6^ human OVCA cells (A2780) harvested from *in vitro* culture. All mice were left untreated for 5–6 days in order for the tumor and its vascular bed to become established. For treatment, the a2v‐mAb dose was selected based on the patented work that establishes the utility of *in vivo* inhibition of the V‐ATPase‐V0a2 activity (previously known as RTF) in the treatment of ovarian carcinoma (US patent number: 7211257; Methods for inducing apoptosis in ovarian carcinoma cells using an antiregeneration and tolerance factor antibody).

Here, after appearance of palpable tumors (3–4 mm^3^), mice were divided into two groups. One group of mice was injected with a2v‐mAb (300 µg antibody in 100 µL PBS; intraperitoneal). In the other group (control), murine IgG isotype antibody (BioXcell, Lebanon, NH, USA) was administered in the same way. The antibody injections were given thrice (total of 900 µg), each dose at 48‐h interval. Mice were weighed every alternate day, starting on the day of palpable tumor appearance. The effect of antibody‐mediated inhibition on tumor growth was carefully monitored by calipers and compared between the test and control groups (*n* = 8 in each). Tumor size was calculated by multiplying [length × 2 (width of the tumor)]/2 on a given day (19). When the tumors grew to a size of 1500 mm^3^ according to the IACUC guidelines, the mice were sacrificed. For evaluating the immune infiltration, the tissues were harvested at day 10 after palpable tumor appearance. The statistical significance of tumor regression was calculated by Student's *t*‐test. All statistical tests were two sided.

Alternately, for testing the efficacy of the antibody dose and route, we used athymic nude mice. The a2v‐mAb efficacy was evaluated (A) by intraperitoneal injection or (B) a2v‐mAb injection in the area of tumor bed or (C) simultaneous a2v‐mAb injection with cancer cells at the time of tumor inoculation.

### 2.4 Immunohistochemical staining

For V‐ATPase‐V0a2 staining in the clinical OVCA tissues, the paraffin‐embedded human OVCA tissue blocks from type 1 and type 2 OVCA patients were obtained from Advocate Lutheran General Hospital (ALGH), Chicago, IL, USA. The study was approved by the Ethics Committee of ALGH. Since the archival samples already available at ALGH were used, this study received a waiver of HIPAA authorization. The study methodologies conformed to the standards set by the Declaration of Helsinki.

Immunohistochemistry was performed using the Dako envision kit (Dako, Carpinteria, CA, USA), according to the manufacturer's instructions. Subcutaneous tumor samples were harvested at 15 mm^3^ size or on the same day (day 10 after palpable tumor appearance) and fixed with 4% formalin (24 h, RT), treated with 30% sucrose (24 h, RT) to cryo‐protect the tissues and then frozen with OCT compound over dry ice. Immunohistochemical staining was performed using anti‐CD45 (Abcam, Cambridge, MA, USA; ab30470), anti‐CD11c (Abcam; ab11029), anti‐F4/80 (Abcam; ab111101), antineutrophil elastase (Abcam; ab68672), anti‐NKR p1c (Abcam; ab174600), anti‐iNOS (Abcam; ab49999), anti‐CA‐125 [muc16)] (Abcam; ab110640), and antiactive caspase‐3 (Abcam; ab2302) antibodies. The tissue sections were stained using a method based on horseradish peroxidase‐labeled polymer (EnVision + Dual Link System‐HRP; DAKO) according to manufacturer's protocol, preceded by an antigen retrieval procedure by boiling the sections in sodium citrate buffer (pH = 6.0). Experiments were performed at least thrice in duplicate. Simultaneously, for negative controls, tissue sections were stained with mouse isotype‐control antibodies (R&D Systems, Minneapolis, MN, USA; catalog no: MAB002) used at the same concentration as the primary antibodies. The sections were counterstained with Mayer's hematoxylin and mounted in Faramount aqueous mounting medium (Dako). The immunostaining was evaluated by light photomicroscopy (Leica ICC50 W, Buffalo Grove, IL, USA) using a high‐resolution camera.

For scoring of immunohistochemical data, the semiquantitative integration method was applied. For this, five random fields of view were observed for each specimen at high magnification (200×). The results were scored based on the following criteria: First, staining area score (SAS; ≤ 1%: 0; 2–25%: 1; 26–50%: 2; 51–75%: 3 and > 75%: 4). Second, staining intensity (SI; light brown: 1; moderate brown: 2 and tan: 3). The Immunohistochemical (IHC) score was calculated using the formula: ICS score = SAS × SI.

### 2.5 RNA isolation and reverse transcription‐PCR

The cells were washed with HBSS (Gibco, Grand Island, NY, USA) and harvested using accutase solution (Sigma‐Aldrich). The harvested cells were washed twice with HBSS by centrifugation at 200 ***g*** for 5 min. RNA isolation was performed using RNeasy® mini kit (Qiagen, Germantown, MD, USA) according to the manufacturer's protocol. Samples were stored at −80 °C until further use. 2.5 µg of total RNA was reverse‐transcribed at 37 °C using random primers and M‐MLV Reverse transcriptase system by high‐capacity cDNA kit (Applied Biosystems, Foster City, CA, USA) as recommended by the manufacturer. At least three biological replicates were prepared for each of the samples. Q‐RT‐PCR was performed using the Step One Real‐Time PCR system (Applied Biosystems), with GAPDH or 18s rRNA as the internal reference. All real‐time PCRs were performed in triplicate in 10 μL volumes using Universal fast PCR Master Mix reagent (Applied Biosystems) according to the manufacturer's protocol. The results were analyzed using the ΔΔ*C*_t_ method.

### 2.6 Targeted RNA‐Seq using next‐generation sequencing

For next‐generation sequencing (NGS), libraries were prepared using Targeted RNA‐seq ‘mouse inflammation and immunity transcriptome’ panel (Qiagen) that contains probes for 485 genes as described earlier [22]. Briefly, cDNA was prepared from 1000 ng of tumor RNA and unique molecular tags of 12 nucleotide length were incorporated into 20 ng cDNA *via* gene‐specific primer extension. After purification, the barcoded cDNA was amplified using gene‐specific primers. The index sequences unique to each sample were inserted in the second PCR. The completed library was loaded into reagent cartridge (150 cycle v3; Illumina, San Diego, CA, USA) and sequenced on a standard flow cell with custom sequencing primers provided by Qiagen. Sequencing quality controls, including cluster density, total reads, and percent reads reaching Q30, were all within optimal ranges provided by Illumina. In addition, secondary quality controls provided by Qiagen's targeted RNA‐Seq software that read and quantify the sequencing files were all within acceptable ranges. The FASTQ files obtained from the sequencing runs were uploaded to Qiagen's Gene Read DNAseq variant calling service. The data were then exported into a format that provides the total unique molecular barcode sequencing reads for each gene. All reads were normalized to 8 internal control housekeeping genes after screening negative for genomic DNA contamination. Student's *t*‐test was performed on the normalized data and expressed as ‘normalized expression’ for the bar diagrams.

### 2.7 Cell survival assay

The exponentially growing ovarian tumor cells were seeded into 96‐well plate (1 × 10^4^/well) overnight. The A2780 cells were treated with a2v‐mAb or control IgG for 48 h at 37 °C in 5% CO_2_. After incubation, *in vitro* cell viability was measured using MTS assay (Promega, Madison, WI, USA) according to the manufacturer's instructions. Experiment was performed in triplicate.

### 2.8 Measurement of the proton (H^+^) pump activity

The proton pump activity was evaluated using the acridine orange (AO) fluorescence quenching method [23]. Human OVCA cell lines A2780 cells were washed twice with Hank's balanced salt solution (HBSS). The cells were resuspended in HBSS at a density of 5 × 10^7^ cells·mL^−1^ and placed on ice. Cells were treated with a2v‐mAb (0, 50, 100, 200, 300 µg) for 30 min at 4 °C. In the control group, cells were treated with Ms IgG antibody at the similar concentrations as the test antibody. OVCA cells (50 µL) were added to prewarmed HBSS (2.5 mL) containing AO (10 µm) and placed in a cuvette. The AO fluorescence was measured at an excitation wavelength of 495 nm and an emission wavelength of 540 nm using the LS‐50B Luminescence Spectrometer (Perkin Elmer, Waltham, MA, USA; Software: FL WinLab, Version 4.00.03). As a positive control, OVCA cells were treated with the bafilomycinA1 (1 µm) for the proton pump activity. A decrease in the fluorescence intensities per min was calculated and expressed as a percentage to the control. Mean ± SD of three independent experiments were used for the analysis.

### 2.9 Immunofluorescence analysis

For immunofluorescence analysis, the tumor tissues from antibody‐treated mice and control mice were used. Frozen tissue sections were boiled in sodium citrate buffer pH 6.0, for 10 min. Upon cooling, the slides were blocked in 3% BSA in PBS for 1 h at RT. For primary antibody incubations, the tissue sections were incubated overnight at 4 °C. The tissue sections were rinsed three times with PBST and then incubated with Alexa Fluor® 488‐conjugated/Alexa Fluor® 594‐conjugated/Alexa Fluor® 405‐conjugated goat/donkey anti‐rabbit/mouse/rat secondary antibody (1 : 200 dilution; Invitrogen) in 1% BSA in PBST. After 45 min of incubation at RT, the tissues were rinsed thrice with PBST. The tissues were mounted using ProLong® Gold (Invitrogen) mounting medium with or without DAPI nuclear stain and allowed to polymerize at room temperature for 24 h. For confocal microscopy, the stained cells were imaged on Olympus Fluoview Fv10i confocal microscope. Analysis was performed using fv10i flouview Ver.3.0 software. Experiments were repeated at least three times in duplicate. For immunofluorescence microscopy, stained cells were imaged in Olympus microscope (Center Valley, PA, USA) and analyzed using NIS‐Elements AR software (Nikon Inc, Melville, NY, USA).

### 2.10 PBMC isolation and coculture experiments

For blood collection and processing, blood from a healthy donor was drawn by peripheral venipuncture into BD vacutainer tubes with sodium heparin (Becton Dickinson, Franklin Lakes, NJ, USA). PBMCs were isolated using Leucosep tubes (Greiner Bio‐One North America, Inc., Monroe, NC, USA ) as per manufacturer's instruction. The isolated PBMCs were washed twice with PBS and then used immediately for the *in vitro* coculture assays.

A2780 human OVCA cells (0.5 × 10^6^) were plated in an apical chamber comprising of a permeable hanging well (0.4 µm pore size) in a 24‐well plate (BD Biosciences, Franklin Lakes, NJ, USA). Upon confluency, the cancer cells were treated with a2v‐mAb (50 and 100 µg·mL^−1^) concentrations in an unbuffered RPMI medium containing 2.5% FCS. After 24‐h treatment of cancer cells, the PBMCs were suspended in the corresponding cell culture medium. 1 × 10^6^ PBMCs (in 50 µL medium) were added to the basolateral chamber of each transwell. Coculture controls consisted of wells containing cancer cells treated with Ms IgG or V‐ATPase inhibitor, bafilomycin. Cocultures were then incubated for 24 h. Following incubation, for RNA isolation from PBMCs, the media containing the PBMCs in the basolateral chamber was transferred to a 1.5‐mL tube and centrifuged to obtain the PBMC pellet. The PBMC pellets were immediately lysed using RLT buffer (RNeasy kit; Qiagen) and processed for RNA extraction.

### 2.11 Antibody‐dependent cell cytotoxicity assay

*In vitro* antibody‐dependent cell cytotoxicity (ADCC) activity of a2v‐mAb was assessed using ADCC Reporter Bioassay kit (Promega) according to manufacturer's instructions.

### 2.12 Statistical analysis

Data were analyzed using GraphPad prism (version 5) statistical software (GraphPad, San Diego, CA, USA). The means of two data sets were compared, and significance was determined by two‐tailed Students *t*‐test or Mann–Whitney test. Differences were considered to be statistically significant where *P* < 0.05. Data are graphically represented as mean ± standard deviation of the mean (SD). All experiments were repeated at least three times in duplicate.

## 3 Results

### 3.1 Vacuolar‐ATPase‐V0a2 is highly expressed in tumors belonging to different morphological subtypes of ovarian cancer and its expression correlates with the tumor grade

**Figure 1:**
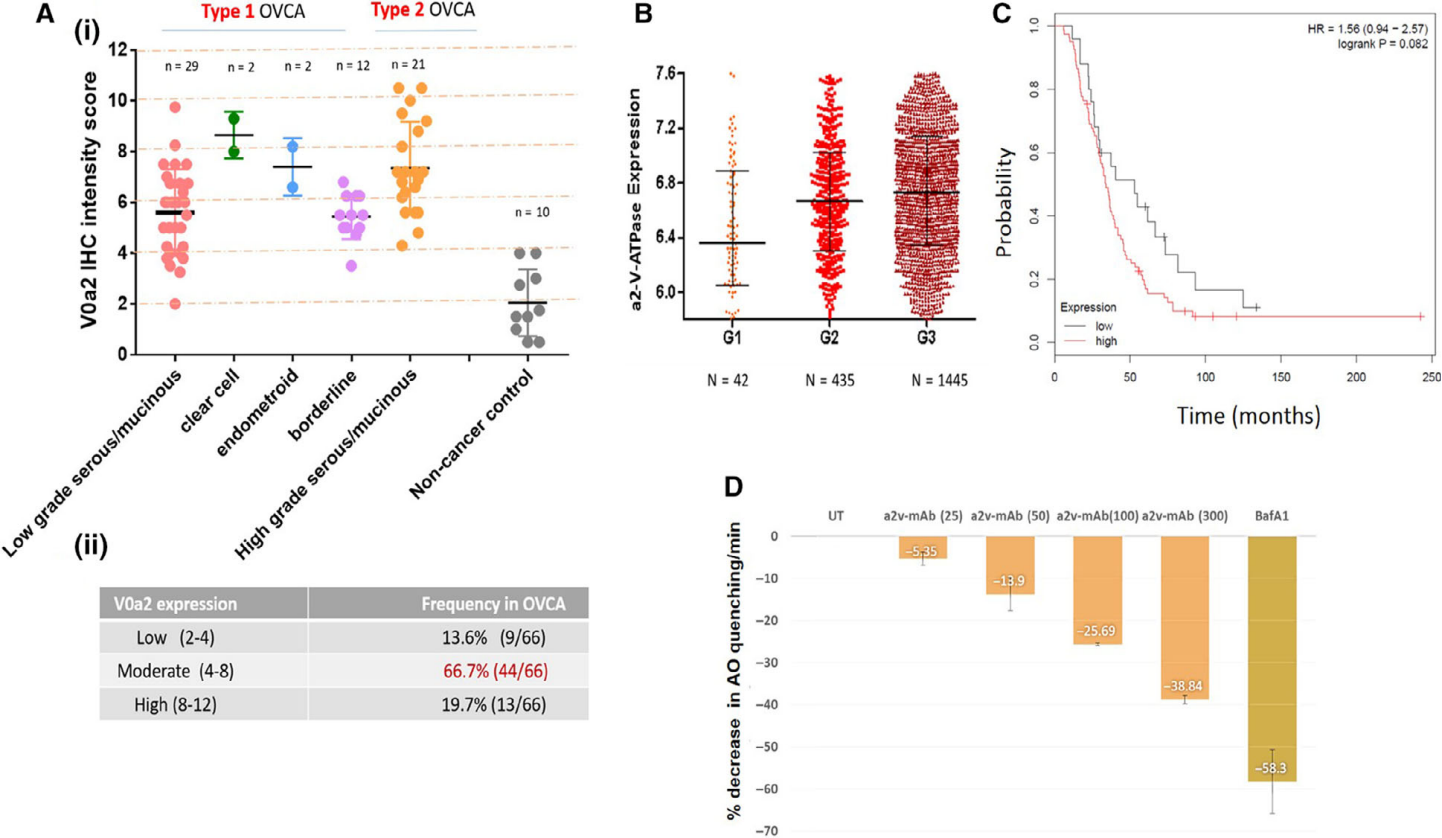
Elevated expression of V‐ATPase‐V0a2 in tumor tissues from patients belonging to different morphological subtypes of OVCA. (A) (i) IHC scoring was performed to evaluate the V‐ATPase‐V0a2 protein expression in type 1 (low‐grade serous/mucinous; *n* = 29, clear cell; *n* = 2 endometrioid; *n* = 2, borderline; *n* = 12) and type 2 (high grade serous/mucinous; *n* = 21) OVCA patients (total *n* = 66). Noncancer control tissue samples (*n* = 10) were used as control. Data represent mean ± standard deviation (SD). Statistical analysis was performed using Mann–Whitney test. The expression of V‐ATPase‐V0a2 was significantly elevated in all morphological subtypes of OVCA compared to nontumor controls. (ii) The table shows the frequency and the intensity of V0a2 expression in OVCA patients. 66.7% of the patients exhibited moderate V0a2 expression based on IHC score. (B) V‐ATPase‐V0a2 gene expression analysis using TCGA database revealed an increasing V0a2 expression with increase in tumor grade (G1; *n* = 42, G2; *n* = 435, G3; *n* = 1445). Graph shows the median values as dark horizontal lines; lower line shows quantile 1 and upper line shows quantile 4. Statistical analysis using Mann–Whitney test. (C) Kaplan–Meier analysis of overall survival and progress‐free survival by low or high ATP6V0a2 expression in OVCA patients who received platinum‐based chemotherapy treatment was performed by using Cox proportional hazard models. (D) Treatment of human OVCA cells with a monoclonal inhibitory antibody (a2v‐mab) against V‐ATPase decreases the proton pumping activity in these cells. For assessing the proton pump activity, the antibody‐treated OVCA cells were evaluated for AO fluorescence quenching in the cell supernatants. Fluorescence intensity at an excitation of 495 nm and an emission of 540 nm was quantified. Decrease in fluorescence intensity per min was calculated as percentage relative to control (mean ± SD; *n* = 3). Chemical V‐ATPase inhibitor, bafilomycinA1, was used as a positive control in the assay. Experiments were repeated three times.

The clinical tumor tissues from 66 cases of OVCA were evaluated. Tumor samples were derived from 45 (68.2%) type 1 OVCA patients (precursor lesions described clearly in the ovary) and 21 (31.8%) type 2 OVCA patients (more aggressive; ovarian origin unclear; tumors may develop de novo). Based on immune‐histochemical analysis, the V‐ATPase‐V0a2 expression was higher (*P* < 0.001) in both type 1 and type 2 OVCA tissues than the noncancer tissues (*n* = 10; Fig. 1A). 66.7% of the OVCA patients exhibited moderate expression of V0a2 in tumor tissues (Fig. 1A, lower table). Bioinformatics analysis of the Cancer Genome Atlas (TCGA) patient data from different OVCA tumor grades (FIGO grade 1 = 2.1%, *n* = 42; grade 2 = 22.6%, *n* = 435; grade 3 = 75.1%, *n* = 1445) indicate that the expression of the ATP6V0A2 gene correlates with OVCA grade (G1 vs G3, *P* = 2.02e‐07; Fig. 1B). Overexpression of V‐ATPase‐V0a2 correlated with poor survival (OS; HR = 1.56; *P* = 0. 082; Fig. 1C). *In vitro* functional analysis established that inhibition of V‐ATPase‐V0a2 using a2v‐mAb inhibited the proton pumping activity in OVCA cells using AO uptake assay (Fig. 1D).

In the human OVCA tissues, we observed that V‐ATPase‐V0a2 is expressed not only on cancer cells, but also on the tumor‐infiltrating immune cells. The V0a2 isoform was abundantly expressed on tumor‐associated macrophages (Fig. S1A,B) and tumor‐associated neutrophils (Fig. S1C,D). This suggests a broader impact of targeting V‐ATPase‐V0a2 in the ovarian TME. These findings indicate that V‐ATPase‐V0a2 is highly expressed in both type 1 and type 2 OVCA and its inhibition impedes V‐ATPase‐mediated proton pump activity.

Next, we examined the effect of monoclonal antibody (a2v‐mAb)‐based V0a2 inhibition on the growth of ovarian tumors in preclinical mouse model.

### 3.2 *In vivo* a2v‐mAb injection enhances immune cell infiltration in tumors and delays ovarian cancer growth

**Figure 2:**
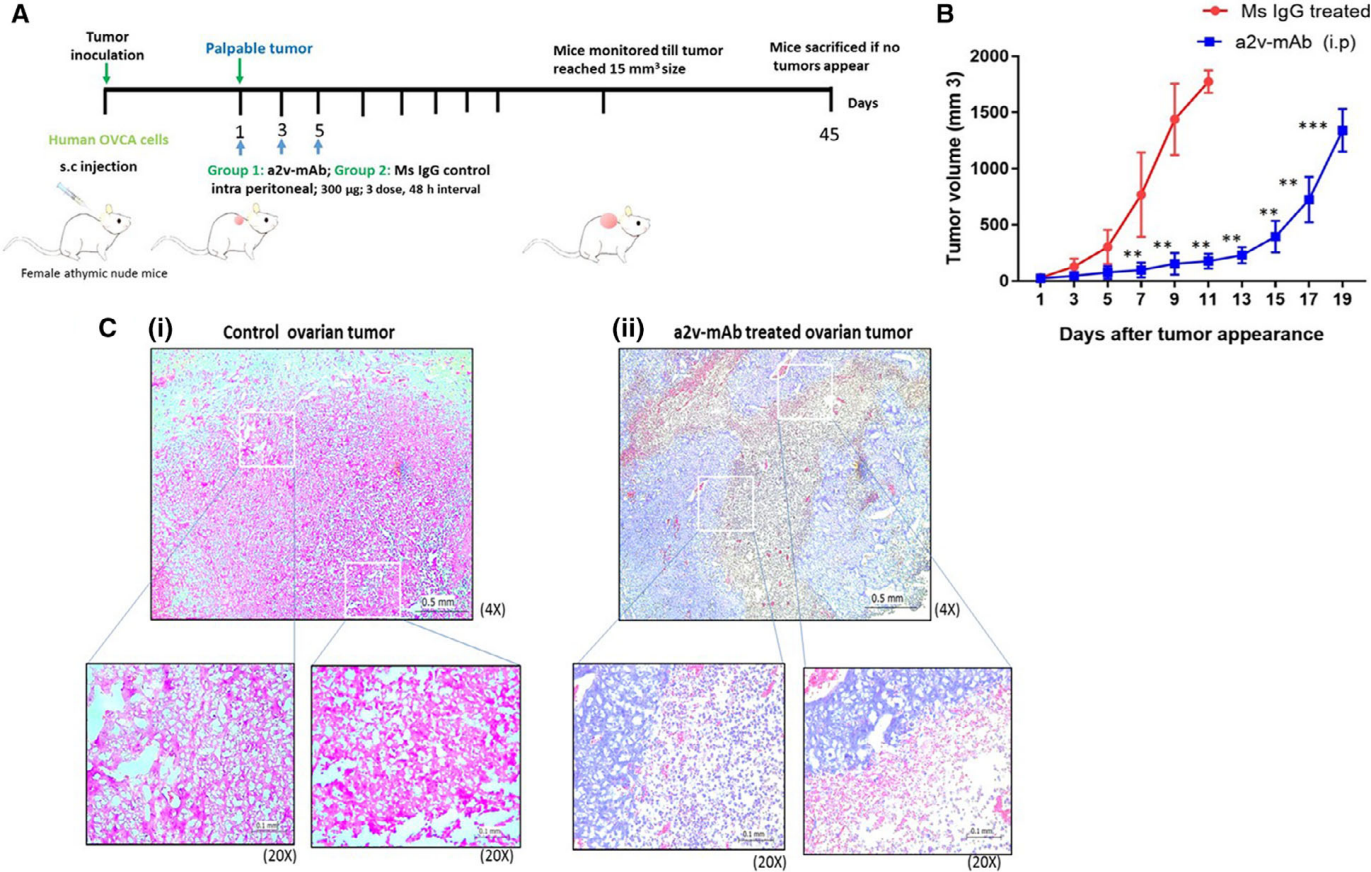
Antibody against V‐ATPase‐V0a2 arrests ovarian tumor growth in athymic nude mice (A) Schematic diagram of the experimental procedure. Anti‐V‐ATPase monoclonal antibody (a2v‐mAb) treatment schedule in OVCA xenograft model. Human OVCA cells (A2780) were implanted subcutaneously into the left upper flank of 3‐to‐4‐week‐old female athymic nude mice. Upon appearance of palpable tumors, intraperitoneal injections of the antibody (300 µg) were given (mouse IgG1 in control group and a2v‐mAb in treatment group (three doses, every alternate day). (B) Tumor volumes were measured by calipers on alternate days right on the first dose of antibody administration. Data are reported as means ± SD (*n* = 8/group) ** p<0.01, *** p<0.001. Two‐way ANOVA analyses were performed. (C) Representative hematoxylin and eosin (H&E) staining of ovarian tumor sections from anti‐V‐ATPase mAb‐treated or the control mice. Compared to (i) mouse IgG‐treated control tumors, (ii) the a2v‐mAb antibody‐treated tumors exhibited high immune infiltration that shows compact solid tumor mass. Magnification 4×; scale bar‐ 0.5 mm. Magnification 20×; scale bar‐ 0.1 mm. The experiment was repeated three times (*n* = 8 in each group).

We evaluated the *in vivo* efficacy of a2v‐mAb treatment in controlling ovarian tumor growth. Human OVCA tumors were generated in female athymic nude mice as described in methods. Upon appearance of palpable tumors, mice were intraperitoneally injected with either a2v‐mAb or mouse IgG isotype control (Fig. 2A). Treatment with a2v‐mAb resulted in a substantial inhibition of tumor growth (*P* < 0.001) compared to the control group (Fig. 2B). No weight loss was observed in the anti‐V‐ATPase mAb‐treated animals (Fig. S2). In addition, a2v‐mAb treatment through different routes of administration also exhibited a delay in OVCA tumor growth in the nude mice model (Fig. S3). The results indicate that V‐ATPase antibody had a significant inhibitory effect on the tumor growth.

To assess the histopathological changes in the xenograft tumors after antibody treatment, we performed H&E staining on the tumor sections. On day 10 after palpable tumor appearance, control tumors exhibited a compact cancer cell bed with limited immune cell infiltration [Fig. 2C(i)]. In contrast, the a2v‐mAb‐treated tumors exhibited larger immune cell infiltrated areas and exhibited a marked decrease in cancer cell‐containing regions [Fig. 2C(ii)].

**Figure 3:**
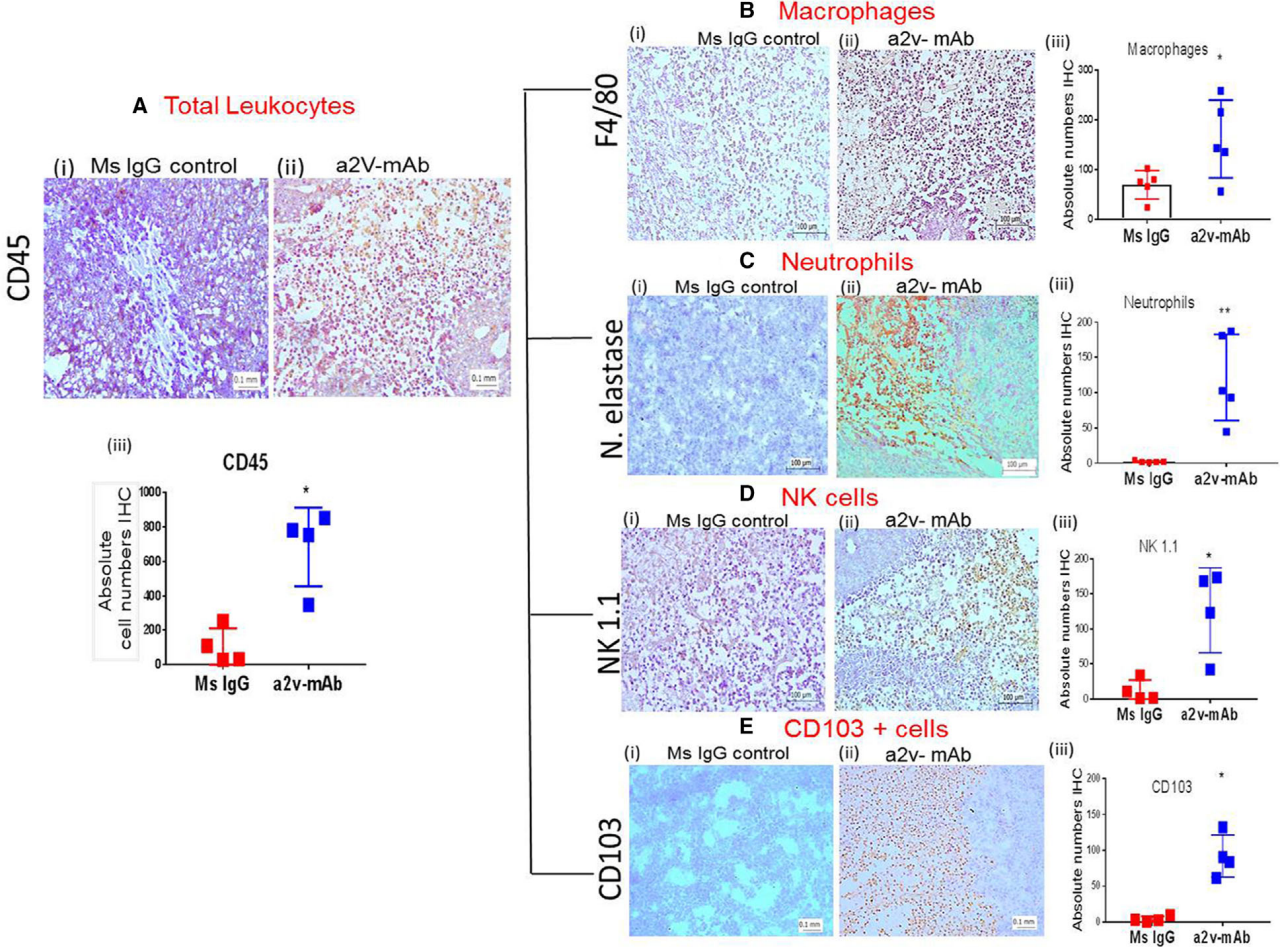
a2v‐mAb treatment increases inflammatory immune population in ovarian tumors that delays tumor growth. (A) Immunohistochemistry analysis of tissue sections from tumors treated with (i) mouse IgG control or (ii) anti‐V‐ATPase antibody (a2v‐mAb) showing anti‐CD45 staining (brown) for total leukocyte population. (iii) The absolute number of CD45‐positive cells was significantly higher in a2v‐mAb‐treated tumor sections compared to control tumors (*n* = 4). (B) IHC analysis of the absolute macrophage population using anti‐F4/80 antibody staining in (i) mouse IgG control or (ii) a2v‐mAb showed significantly upregulated numbers of F4/80 cells in antibody‐treated tumors (*n* = 5). (iii) Bar graph showing the difference in the absolute macrophage numbers in antibody‐treated vs control tumors. (C) IHC analysis of the neutrophil population using antineutrophil elastase (N. elastase) antibody staining in (i) mouse IgG control and (ii) a2v‐mAb‐treated tumors (*n* = 5). (iii) Bar graph showing the difference in the absolute neutrophil numbers in antibody‐treated vs control tumors. (D) IHC analysis of the NK cell population using anti‐Nk1.1 antibody staining in (i) mouse IgG control or (ii) a2v‐mAb‐treated tumor sections (*n* = 4) showed significantly upregulated numbers of NK cells in antibody‐treated tumors. (iii) Bar graph showing the difference in the NK cell population in antibody‐treated vs control tumors. (E) IHC analysis of the CD103^+ ^cell population using anti‐CD103 antibody staining in (i) Mouse IgG control or (ii) a2v‐mAb‐treated tumor sections showed significantly upregulated numbers of CD103^+ ^cells in antibody‐treated tumors (*n* = 4). (iii) Bar graph showing the difference in the CD103^+ ^cell population in antibody‐treated vs control tumors. Experiments were repeated at least thrice in duplicate. Images at 20× magnification; scale bar‐ 0.1 mm. Bar graph data represented as mean ± SD; statistical analysis using Mann–Whitney test, *P < 0.05.

We further evaluated the immune cell subtypes that are altered in the a2v‐mAb‐treated tumors by immune‐histochemical analysis. Intratumoral immune cells in the antibody‐treated mice contained abundant leukocyte population (CD45; Fig. 3A) compared to the control mice (*P* = 0.028). The immune cell populations of these tumors showed an increase in the total macrophage population (F4/80, *P* = 0.038; Fig. 3B) as well as neutrophils (neutrophil elastase, *P* = 0.0024; Fig. 3C), NK cells (NK1.1, *P* = 0.028; Fig. 3D), and CD103^+ ^cells (mainly dendritic cells in nude mice; CD103, Fig. 3E, *P* < 0.05) in the a2v‐mAb‐treated nude mice.

### 3.3 Tumors of a2V‐mAb‐treated mice develop an antitumorigenic environment by the expression of macrophages secreting iNOs

**Figure 4:**
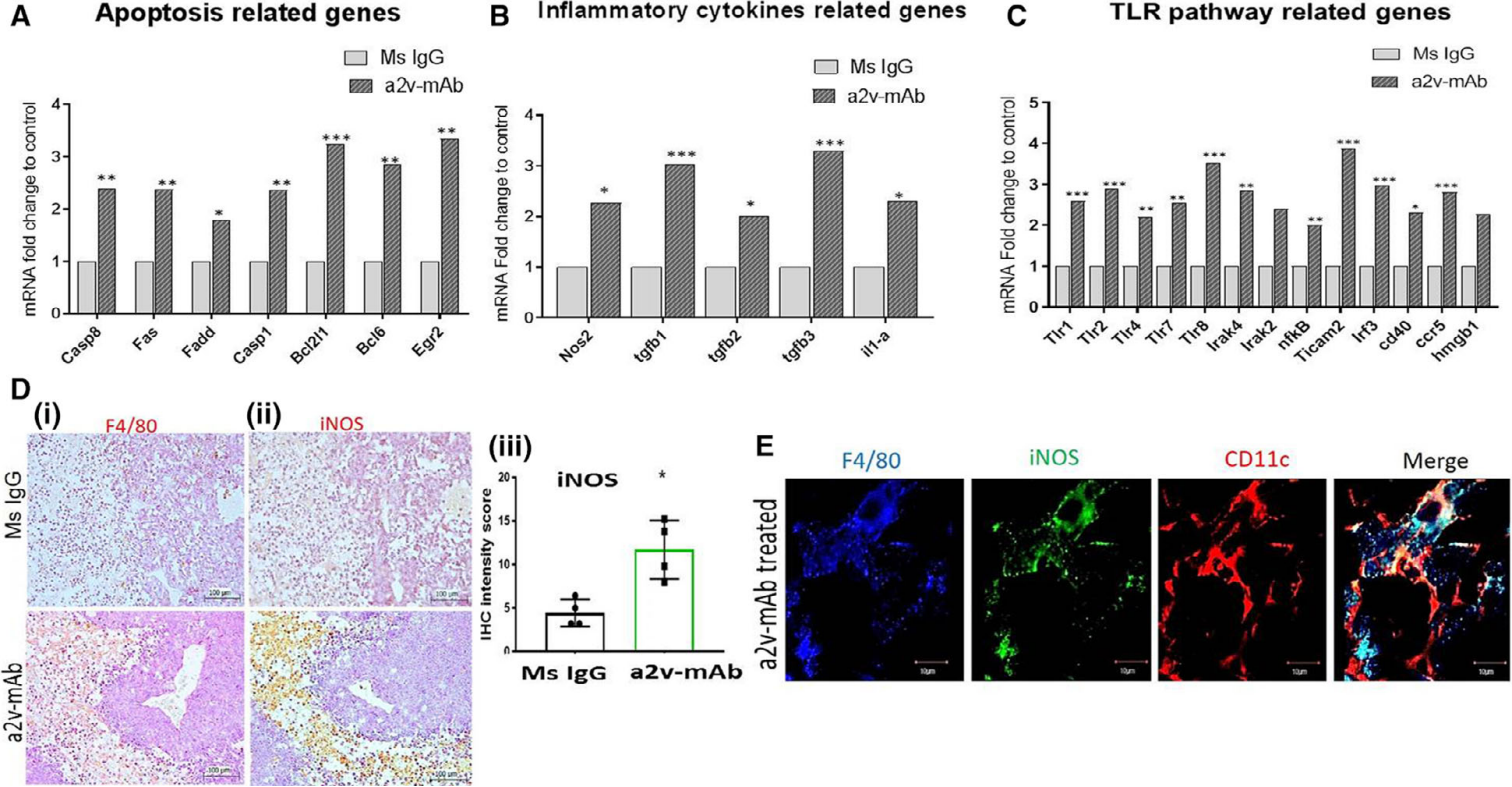
a2v‐mAb‐treated tumors overexpress TLR pathway and inducible nitric oxide that supports antitumor response. Equal amounts of tumor RNA was used for targeted RNA‐Seq (Miseq), and data were analyzed online using Qiagen's data analysis center geNorm (Entire Gene Panel) method that utilizes multiple reference genes was used to normalize the gene expression. Bar graphs show fold change transcript levels of (A) apoptosis‐related genes, (B) inflammatory cytokine‐related genes, and (C) TLR pathway‐associated genes in a2V‐mAb TME compared to control tumors. Average mean data from a2v‐mAb‐treated; *n* = 4 and control; *n* = 5 mice are depicted here. For gene alteration, twofold cutoff value was applied. Genes with significant alteration are shown as *P < 0.05, **P < 0.01, ***P < 0.001 using Student's *t*‐test. (D)Immunohistochemical analysis of the serial sections from ovarian tumors treated with a2v‐mAb or Mouse IgG antibody showing a significantly enhanced expression of macrophages [(i) F4/80 staining and (ii) iNOS expression in the same tumor areas in a2v‐mAb‐treated tumors compared to control]. Magnification‐20×; scale bar‐100 µm. (iii) Bar graph showing a significantly increased expression of iNOS in a2v‐mAb‐treated tumors compared to Ms IgG controls, mean ± SD; Mann–Whitney test (*n* = 4/group). (E) Confocal microscopy analysis showing that the macrophages (F4/80; red) expressing iNOS ( green) are M1 phenotype (CD11c; blue). Merged areas in white show coexpression. Scale bar‐10 µm. Experiments were repeated thrice in duplicate.

We performed targeted RNA‐Seq *via* NGS to determine the altered immune profile of tumors from a2v‐mAb‐treated mice. The data show that transcripts of 140 out of 485 genes analyzed were altered by more than twofold in a2v‐mAb‐treated tumors (*P* < 0.05; Fig. S4). A total of 134 genes were significantly upregulated while 6 genes were significantly downregulated (Fig. S4). Among the genes analyzed in the a2v‐mAb tumors (Fig. 4A), apoptosis‐related molecules including caspase 8, Fas, Egr2, and Fadd molecules were elevated (*P* < 0.05). Among antiapoptotic molecules, bcl6 and bcl2L were upregulated. The transcripts of inflammatory cytokines, including Nos2 (2.28‐fold, 0.037557), interleukin (IL)‐1α (2.3‐fold, *P* = 0.025), TGF β 1 (3.03‐fold, *P* = 0.0005), TGFβ 2 (twofold, *P* = 0.028), TGF β 3 (3.34‐fold, *P* = 0.007), were upregulated in the a2V‐mAb‐treated TME (Fig. 4B). Elevated expression of high‐mobility group box 1 (HMGB‐1) that stimulates interferon‐gamma (IFN‐γ)‐producing Th1 response in immune cells was also observed (2.28‐fold). Several TLRs including the transcripts of Tlr4 (2.21‐fold, *P* = 0.002), Tlr8 (3.53‐fold, *P* = 0.0008), Tlr2 (2.91‐fold, *P* = 0.0009), Tlr1 (2.59‐fold, *P* = 0.0003), and Tlr7 (2.56‐fold, *P* = 0.005) were elevated (Fig. 4C). CXCl‐9, the chemokine that mediates lymphocytic infiltration to the focal sites and suppresses tumor growth, also showed a trend toward upregulated expression (10.1‐fold, *P* = 0.09). TICAM‐2 and IRF‐3, downstream signaling molecules of TLR‐4 pathway that activate IFN mediated Th1 responses, were upregulated. CD40, a TNF receptor on DCs that promotes antitumor immunity, was elevated (2.31‐fold, *P* = 0.02). Elevated levels of Fos were observed (5.73‐fold, *P* = 0.011) in the a2v‐mAb TME. Several pro‐inflammatory cytokines genes were also upregulated that included Nfkβ (2.00‐fold, *P* = 0.019) and STAT3 (2.11‐fold, *P* = 0.0018). Q‐RT‐PCR analysis confirmed upregulated expression of IFN‐y (*P* = 0.0079) in the a2V‐mAb‐treated TME (Fig. S5).

F4/80 is a marker for the macrophage lineage. iNOS is a marker for M1 macrophages. We found that an increased number of macrophages in the a2v‐mAb TME express increased levels of iNOS, which is a hallmark antitumor macrophages (Fig. 4Di,iii). The iNOS protein expression was 2.8‐fold higher in a2v‐mAb TME (Fig. 4Diii). F4/80, CD‐11C, and iNOS coexpression was further validated by immune‐fluorescence analysis (Fig. 4E). The results confirm the predominance of M1 macrophages secreting antitumor cytokine, iNOS, in the a2v‐mAb TME that results in delayed tumor growth.

### 3.4 a2v‐mAb‐treated ovarian tumors exhibit low cancer cell numbers and high caspase‐3 expression

**Figure 5:**
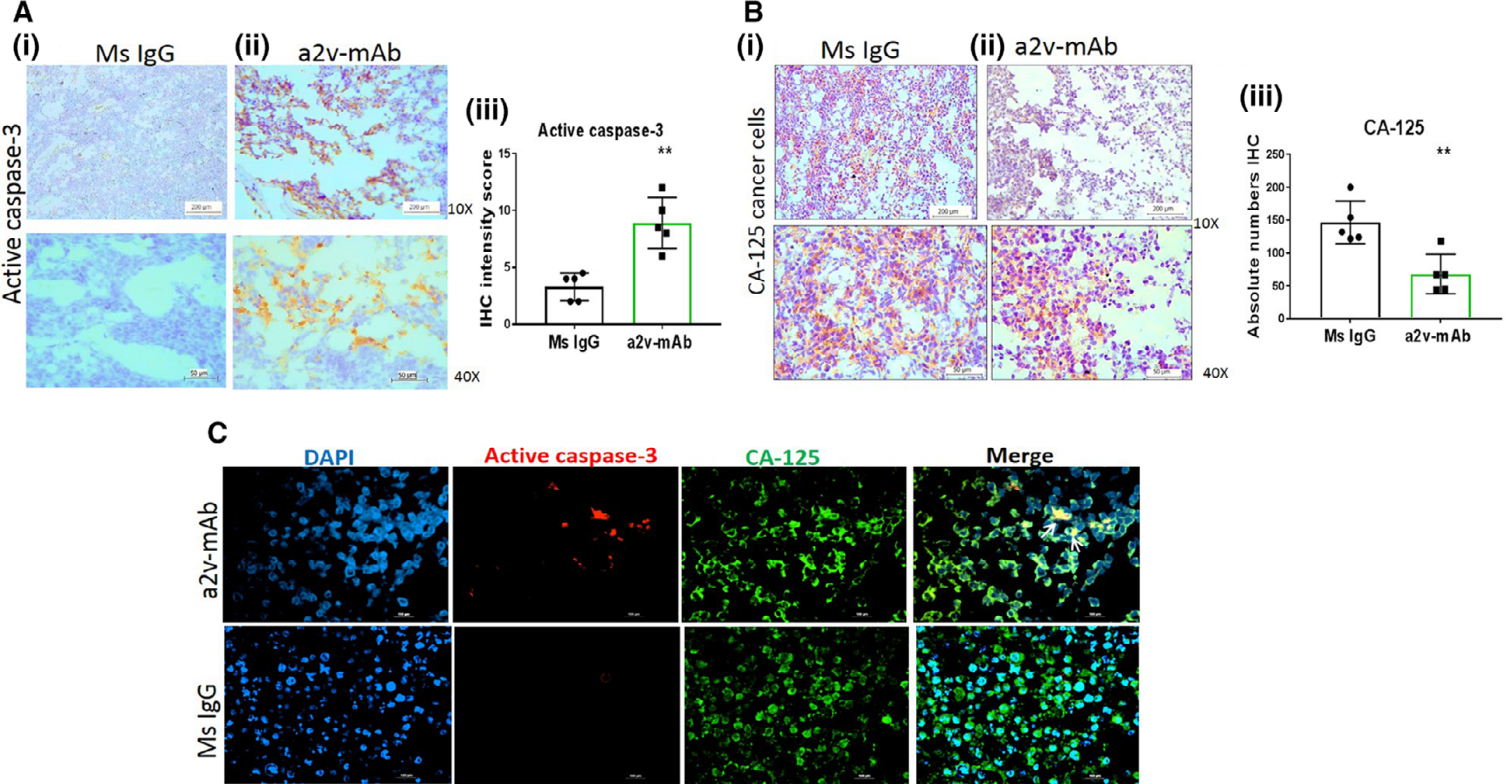
a2v‐mAb‐treated ovarian tumors exhibit low cancer cell numbers and high caspase‐3 expression. (A) Immunohistochemistry analysis of tissue sections from tumors treated with (i) control mouse IgG antibody or (ii) a2v‐mAb showing active caspase‐3 staining (brown) for apoptotic cells at magnification 10× (scale bar‐200 µm) and 40× (scale bar‐50 µm). (iii) Bar graph shows that a2v‐mAb‐treated tumors displayed significantly high caspase‐3‐positive cells compared to control tumors depicted as mean ± SD (*n* = 5/group). (B) Immunohistochemistry analysis of tissue sections from tumors treated with (i) control mouse IgG antibody or (ii) a2v‐mAb antibody showing CA‐125 staining (brown) for cancer cells at magnification 10× (scale bar‐ 200 µm) and 40× (scale bar‐50 µm). (iii) a2v‐mAb‐treated tumors displayed significantly decreased CA‐125‐positive cells compared to control tumors depicted as mean ± SD (*n* = 5/group). (C) Immunofluorescence analysis showing a2v‐mAb‐treated tumor tissues expressing active caspase 3 (red) in cancer cells (CA125; green). Merged areas in yellow depict coexpression; scale bar‐100 µm. Experiments were repeated three times. Statistical analysis using Mann–Whitney test; *p<0.05, **p<0.01.

Immunohistochemistry of a2v‐mAb‐treated tumors showed a significant increase in active caspase‐3 staining (Fig. 5A) and a decrease in CA‐125 OVCA cell staining (Fig. 5B). Quantification of CA‐125 and active caspase‐3‐positive cells revealed that a2v‐mAb treatment increases tumor cell death (active caspase‐3) by 2.8‐fold within antibody‐treated tumors and reduces the ovarian tumor growth (CA‐125‐positive cells; *P* < 0.05) by 3.5‐fold (Fig. 5Aiii,Biii). To confirm the cancer cell death, the costaining of CA‐125 and caspase‐3 marker in the tumor sections was performed using immune‐fluorescence analysis (Fig. 5C). These results confirm that a2v‐mAb significantly reduces the cancer cell numbers in ovarian tumors.

**Figure 6:**
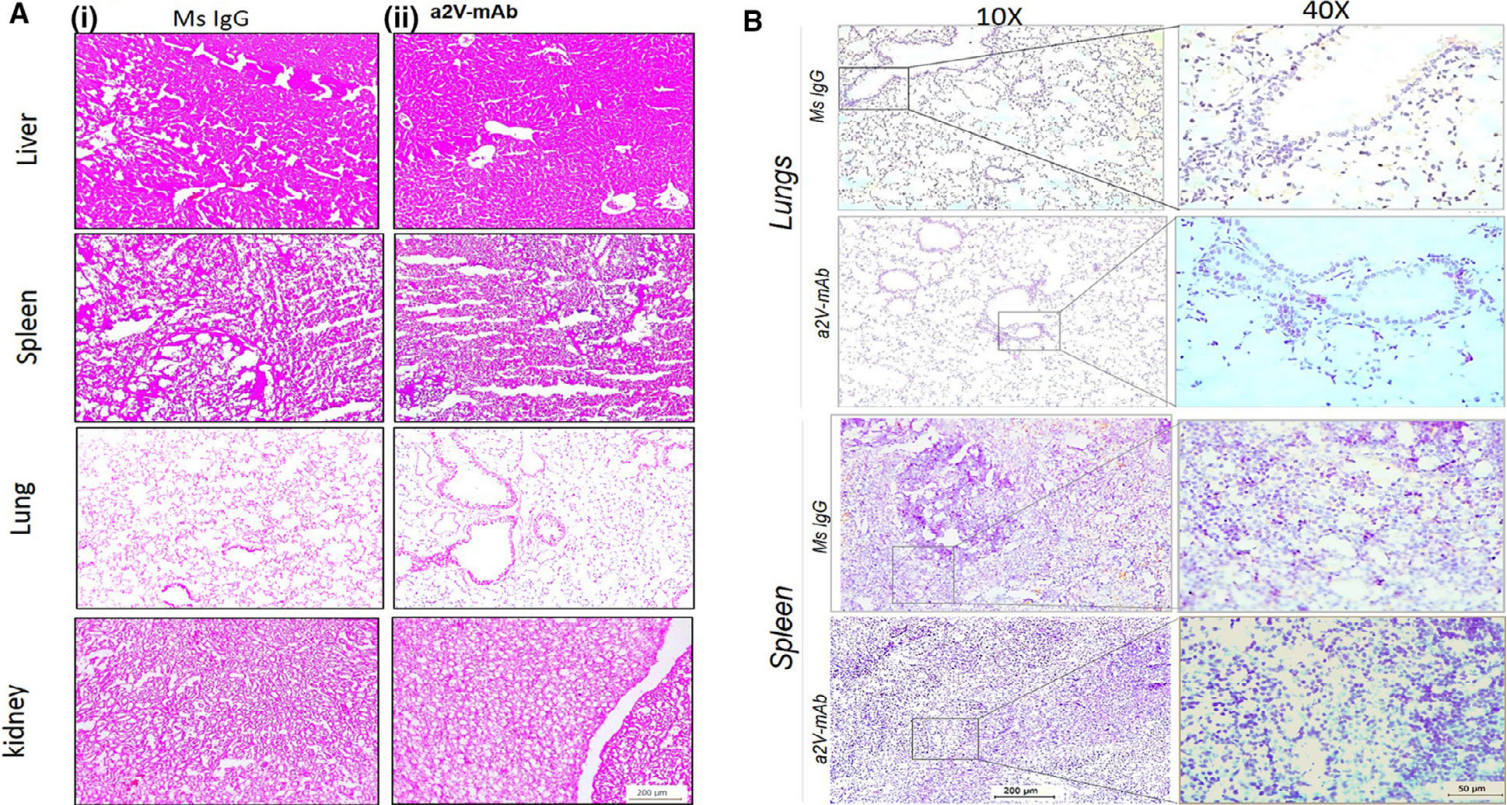
a2v‐mAb treatment does not cause *in vivo* cytotoxicity or metastasis to OVCA bearing nude mice. (A) H&E staining of the liver, spleen, lung, and kidney of the mice treated with (i) control mouse IgG antibody or (ii) a2v‐mAb antibody treatment at 10X magnification (scale bar‐200 µm). a2v‐mAb‐treated mice retained the normal histological features of the vital organs similar to control mice. The experiments were repeated three times. (B) Representative histopathology images of CA125 staining for OVCA cells in mouse lung and spleen sections from control and a2V‐mAb‐treated mice. Magnification 10× (scale bar‐200 µm) and 40× (scale bar‐50 µm). Experiments were repeated thrice in duplicate. *n* = 4 mice were used for analysis.

Further, we observed that the a2v‐mAb treatment did not cause *in vivo* toxicity to OVCA bearing nude mice. No histopathological changes in liver, spleen, lung, and kidney were found in the H& E‐stained tissue sections from these organs (Fig. 6A) and no metastasis was observed in a2v‐mAb mice spleen and lungs (Fig. 6B). Treatment resulted in no adverse effects in terms of gross weight (Fig. S2).

Antibody‐dependent cell cytotoxicity is a mechanism for killing target cancer cells using IgG antibody‐based drugs. The antibody binds to target antigens on the cell surface. When the Fc effector portion of target‐bound antibodies also binds to FcγRIIIa receptors on the cell surface of effector cells (T cells, NK cells), multiple cross‐linking of the two cell types occurs, leading to pathway activation of ADCC. *In vitro* treatment of target cells (cancer cells, B cells) with a2v‐mAb activated the NFAT pathway (expressing luciferase) in the effector T cells that causes target cell death. The a2v‐mAB showed a low ADCC activity (Fig. S6). The direct treatment of a2v‐mAb did not cause cytotoxicity to OVCA cells (Fig. S7).

### 3.5 PBMCs exposed to a2v‐mAb‐treated ovarian cancer cells highly express pro‐inflammatory cytokines

**Figure 7:**
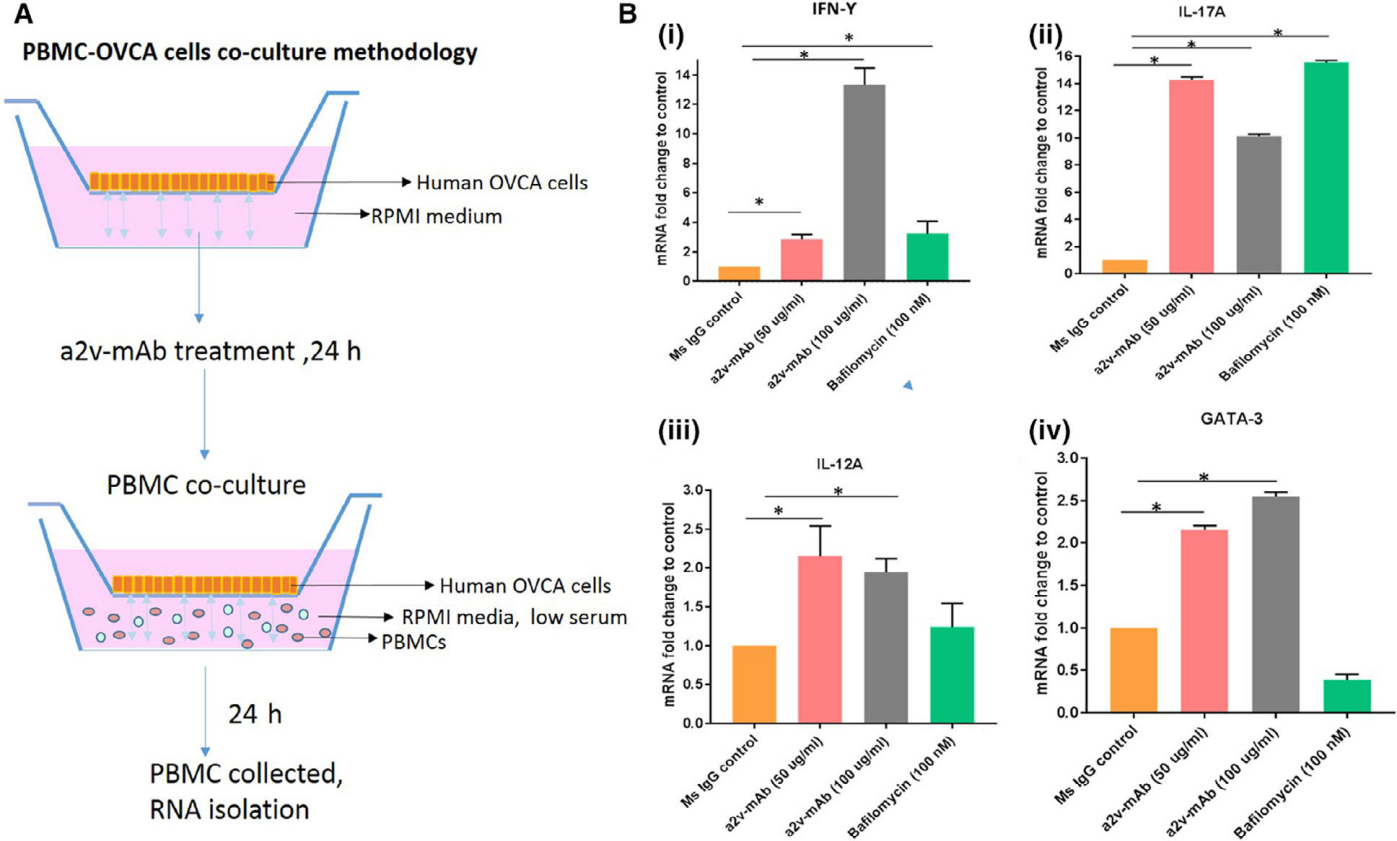
Conditioned media from a2v‐mAb‐treated ovarian cancer cells enhances gene expression of pro‐inflammatory cytokines in PBMCs. (A) Human OVCA cell line (A2780) was cultured in the apical wells of the hanging well inserts. Upon reaching confluency, OVCA cells were treated with a2v‐mAb, Ms IgG, V‐ATPase inhibitor bafilomycin, or with blank media (low serum, unbuffered RPMI medium) for 24 h. PBMCs were isolated and cultured in the conditioned media for 24 h in the presence of the treated ovarian cancer cells. Total RNA was isolated from PBMCs, and gene expression of selected cytokines genes was assessed by RT‐qPCR. (B) Gene expression of (i). IFN‐g and (ii) IL‐12 associated with Th1 response and IL‐17, associated with Th‐17 response in PBMCs was found upregulated. Statistical analysis using Mann–Whitney test, *P < 0.05. (iv) mRNA expression of GATA‐3, associated with Th2 response in PBMCs, was also found elevated. Data are presented as mean and standard deviations of three replicates with normalized expression using 18s rRNA as endogeneous control.

To determine the cumulative impact of conditioned media (CM) from a2v‐mAb‐treated cancer cells on lymphocytes and monocytes, PBMCs were cultured in the presence of CM from a2v‐mAb‐treated OVCA cells (Fig. 7A). The representative Th1/Th2‐cytokines were analyzed by Q‐RT‐PCR. The gene expression of IFN‐y and IL‐12, associated with Th1 response, was found elevated (*P* < 0.05; Fig. 7Bi,ii). IFN‐γ is an effector molecule with associated antiproliferative, pro‐apoptotic, and antitumor mechanisms [24]. IL‐12 is one of the most potent cytokines in mediating antitumor activity [25]. Th17 is the most antitumoral phenotype of T cells [26]. IL‐17, a pro‐inflammatory cytokine produced by Th17 cells, contributes to antitumor immunity by promoting an antitumor cytotoxic T‐cell response leading to tumor regression [27]. Here, IL‐17, associated with Th‐17 response in PBMCs, was markedly increased in the PBMCs after exposure to CM from a2v‐mAb‐treated OVCA cells in the coculture experiments (*P* < 0.05; Fig. 7Biii]. These findings indicate an immune‐stimulating effect caused by exposure of PBMCs to a2v‐mAb‐treated OVCA cell‐conditioned medium, characterized by the upregulation of pro‐inflammatory cytokines. Interestingly, the mRNA expression of GATA‐3, associated with Th2 response in PBMCs, was also found elevated (*P* < 0.05; Fig. 7Biv).

The results suggest that a2v‐mAb treatment delays ovarian tumor growth by increasing the antitumor immune responses, the mechanism that can improve the responses to current therapeutic options including the ICB therapies.

## 4 Discussion

**Figure 8:**
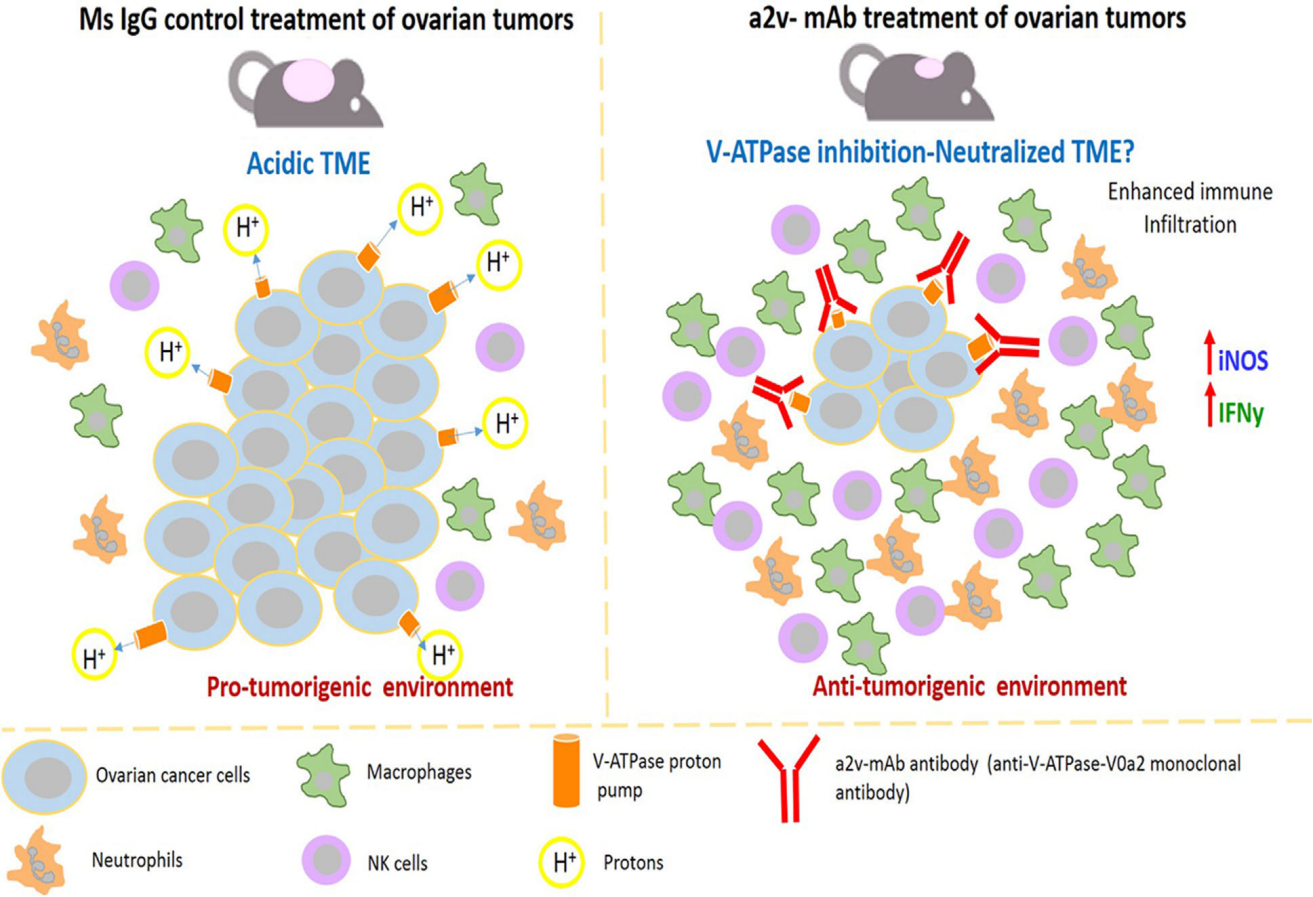
Delayed ovarian tumor growth by V‐ATPase monoclonal antibody treatment is due to altered immune landscape that favors antitumor response. The acidic TME in the ovarian control tumors results in an immune‐suppressive microenvironment with low numbers of innate immune cells. This results in increased ovarian tumor growth. The ovarian tumor‐bearing mice treated with anti‐V‐ATPase‐V0a2 monoclonal antibody (a2V‐mAb), known to have a proton pump inhibition activity, results in an increased infiltration of immune cells including M1 macrophages, NK cells, and neutrophils, which results in drastic increase in antitumor response that involves enhanced iNOS and IFN‐Ƴ expression in the TME of a2v‐mAb‐treated mice. This antitumorigenic microenvironment is conducive for reduced ovarian tumor growth in a2v‐mAb‐treated mice.

Ovarian cancer is the most lethal gynecologic malignancy in the Western world [28]. New molecular‐targeted therapies show promise in managing the late‐stage disease and marginally improving the 5‐year survival rates in OVCA patients [29]. Strategies targeting a centralized physiological mechanism hold promise in improving the treatment outcome across all the morphological subtypes of OVCA. Here, we have targeted a specific plasma membrane‐associated isoform of V‐ATPase that is uniquely expressed on the surface of cancer cells and tumor‐associated leukocytes. This study reports for the first time, the *in vivo* therapeutic efficacy of a proton pump inhibiting antibody (a2v‐mAB) targeting specific ‘V‐ATPase‐V0a2’ surface isoform, in controlling ovarian tumor growth. Tumors from a2v‐mAb‐treated mice displayed high immune cell infiltration and an enhanced antitumor response that delayed the tumor growth, making it an effective treatment approach against OVCA (Fig. 8).

The pH dysregulation due to Warburg effect is among the critical physiological adaptations in cancer cells. The altered pH gradient modulates the cellular signaling and provides survival benefits for cancer cell growth [30]. Additionally, the excess acid is thrown out of the cells making the TME acidic. The acidic TME contributes to an enhanced metastasis, by activating degradative proteases as well as interfering with drug uptake [31]. The microenvironmental acidity also regulates the diverse components of tumor immune surveillance [8]. Eventually, this aids in immune escape and cancer spread. Manipulation of the TME pH has a considerable potential in cancer therapy [7, 8].

Vacuolar‐ATPases, the proton extruding nanomotors on the cancer cell surface, are excellent targets for anticancer agents [32]. Since the highly metastatic cells preferentially use plasma membrane proton pumps to acidify the TME, strategies selectively targeting these surface V‐ATPases hold promise to control tumor progression and successfully cure cancer [33]. However, the toxicity of many of anti‐V‐ATPase agents such as bafilomycinA1 and concanamycin to normal cells makes them a prohibited candidate for use.

*In vivo* V‐ATPase inhibition using small interfering RNA or chemical proton pump inhibitors (PPI) such as omeprazole led to marked inhibition of human tumor growth in xenograft model [12]. In context of a2v‐mAb as V‐ATPase inhibitor, previous studies with this antibody demonstrate its ability to slow the cancer cell migration as well as sensitizing the cisplatin‐resistant cells to treatment [16, 34]. Here, we establish for the first time, the *in vivo* role of a2v‐mAb in delaying ovarian tumor growth in preclinical mouse model. The a2v‐mAb enhances immune cell infiltration in ovarian tumors that support antitumor responses such as increased iNOS expression, leading to delayed tumor growth.

Multiple innate and adaptive immune effector cells and molecules play a role in recognition and destruction of cancer cells by the mechanism called immune surveillance. In OVCA patients, the abundance of tumor‐infiltrating lymphocytes is associated with improved survival [35]. Limited infiltration of NK cells in primary ovarian tumors skews the immune responses toward a suppressive phenotype. The immune cell proportions, heterogeneity, and their spatial distribution hugely impact the tumor immune microenvironment. The cancer cells escape this method by selection of poorly immunogenic tumor cells that modulate cytokine expression of TME recruited immune cells and cause immune suppression. The significance of modulating such cytokines in favor of antitumor response using TME pH modulators will help overcome pro‐tumorigenic potential of cytokines in the TME.

Tumor acidosis suppresses the effective antitumor immune responses [6]. Intratumoral cytokines secreted by infiltrated immune cells are the key mediators of the complex pattern of immune responses that can either aid in cancer progression or to eliminate it [36]. The infiltrated tumor‐associated macrophages can be reprogrammed by the low pH of TME that inhibits iNOS activity and exert tumor proliferative and pro‐angiogenic effects by inducing VEGF and MMP production [37].

Previously, the presence of PPI led to the alteration of TME pH and a reduction of tumor growth and metastasis [38, 39]. The role of V‐ATPase‐V0a2 isoform has already been established in breast cancer, where the shRNA‐mediated targeting of V0a2 on tumor cells has been shown to alter the host immune response that controls tumor growth [19]. In breast cancer, macrophages cocultivated with V‐ATPase knockdown cancer cells produce lower amounts of tumorigenic factors *in vitro* and have a reduced ability to suppress T‐cell activation and proliferation compared with control cells. Delayed mammary tumor growth was observed in Balb/c mice inoculated with V‐ATPase‐V0a2 knockdown breast cancer cells. This was shown to be due to an increase in the M1 macrophage population [19]. The present study reveals that there is an altered immune landscape in a2v‐mAb‐treated ovarian tumors due to an enhanced antitumor immune response, that is able to contain the tumor growth.

Toll‐like receptors play critical roles in the initiation of innate and adaptive immune responses. Danger‐associated molecular patterns, such as HMGB‐1, bind to TLRs, particularly TLR4 on the dendritic cells, causing an inflammatory responses that provides DCs with danger signals, and thereby, stimulating antitumor T‐cell response [40, 41, 42]. TLR4 is expressed not only on tumor cells but also on stromal cells and immune cells that play vital role in antitumor in the TME [40, 43]. Accumulating evidences demonstrates that the activation of TLR4 in the TME can not only boost the antitumor immunity but also give rise to immune surveillance [42]. Loss of TLR‐4 function in cancer patients has been associated with a rapid relapse after chemotherapy than the patients with normal TLR4 expression [44]. In addition, CD40 receptors on the DCs promote antitumor T‐cell activation and re‐educate macrophages to destroy tumor stroma [45]. CD40 activation is a critical mechanism to convert so‐called cold tumors to hot ones (high T‐cell infiltration) that sensitizes them to checkpoint inhibition. Antitumor activity of the agonist CD40 antibodies has been observed in patients with melanoma. Moreover, the destruction of stroma by CD40‐activated macrophages may enhance chemotherapy delivery [45]. Here, we observed a significant increase in CD40 transcript expression in the tumors of a2v‐mAb‐treated mice, suggesting an enhanced macrophage‐mediated antitumor activity.

The high expression of the iNOS is the hallmark of M1/antitumor macrophages [46]. The high levels of NO generation by macrophages effectively induce cell cycle arrest and apoptosis, eventually leading to tumor cell death [47]. In OVCA, low iNOS expression in the clinical tissues correlates with poor prognosis [48]. It is well documented that nitric oxide (NO) is an effector molecule of macrophage‐mediated tumor cell toxicity *in vivo* [49]. The tumor rejection in tumor‐preimmunized mice is associated with a local upregulation of NO synthase (NOS) [50]. Reduced NO synthesis in mice by a NOS inhibitor increases the cancer growth [51]. Here, an increased mRNA levels of NOS‐2 suggest an increase in tumor‐infiltrated immune population that is a source of this NOS‐2 that plays a role in delaying the ovarian tumor growth.

Among the antitumor cytokines, IFN‐γ forms the basis of an extrinsic tumor suppressor mechanism in immunocompetent hosts [52]. The progressive loss of IFN‐γ production by NK cells in cancer is ascribed to decreased pH values and lactate accumulation in the microenvironment of growing tumors [53]. The mechanisms of IFN‐y‐mediated tumor control include inhibition of angiogenesis and the induction of senescence in tumor cells. Increased levels of IFN‐y enhance the recruitment of NK cells to the tumor site that controls tumor growth [54]. Here, we observed a similar trend with ovarian tumors showing an increased NK cell recruitment and a high IFN‐y production upon anti‐a2v mAb administration that contributes to controlling the tumor growth.

The role of TGF‐β in cancer is paradoxical and is stage specific. Specifically in early carcinogenesis, TGF‐β acts as a tumor suppressor where it inhibits cell cycle progression and promotes apoptosis [55]. TGF‐β is an effective inhibitor of cellular growth and deficient TGF‐β pathway can result in unrestrained proliferation leading to tumor development. TGF‐β inhibits the growth of OVCA cell lines *in vitro* [56]. Loss of TGF‐β signaling promotes squamous cell carcinomas by inducing Ras mutations and apoptosis reduction, suggesting that a deficient TGF‐β pathway contributes to tumorigenesis [57]. Here, we observed an increase in TGF‐β transcripts in a2v‐mAb tumors that may suggest its role as antitumor molecule in OVCA.

A Th2 type inflammation at the tumor site facilitates carcinogenesis and tumor progression. The sustained immunological shift from a Th2 to Th1 response is central for an anticancer treatment to be effective. However, studies have found that a sustained Th1 shift is harder to achieve as the tumor load increases. The anticancer mAb induced Th1 response declines within weeks after mAb administration [58]. Our results bolster the idea that there is an elevated anti‐inflammatory Th1 response in the a2v‐mAb‐treated tumors compared to control. This delays the tumor growth; however, as reflected from a concomitant presence of Th2 responses, a complete tumor regression is not achieved. Further investigation is required to demonstrate whether prolonged treatment with the a2v‐mAb can overcome this problem and sustained Th1 cytotoxic responses can be attained.

Since the athymic nude mice are unable to reflect the effect of a2v‐mAb treatment on adaptive immune cells, we cultured the PBMCs with antibody‐treated cancer cells in an unbuffered medium *in vitro*. This takes into account (a) the direct effect of V‐ATPase inhibition on cell culture pH as well as (b) changes in secretion of soluble factors from cancer cells that impact PBMCs. The gene expression of key determinants of Th1 and Th17 response was found elevated, indicating that the a2v‐mAb‐treated cancer cells contribute to elicit a pro‐inflammatory, antitumor immune response that delays cancer growth. Although the Th2 molecules were also enhanced, the immune‐activating response was greater than the immune‐suppressive immune response, as depicted by suppressed tumor growth.

IgG1 antibodies stimulate ADCC [59]. Several anticancer therapeutic mAbs have the IgG1 backbone and are shown to stimulate ADCC which includes trastuzumab [an anti‐(EGFR) 2 (HER2) mAb], rituximab [anti‐(CD)20 mAb], and cetuximab (an anti‐EGFR mAb). Combining immune checkpoint inhibitors (ICIs) with IgG1 antibodies will help overcome the immunosuppression in the TME [59]. The a2v‐mAB is an IgG1 antibody targeted against the tumor cell surface is a suitable candidate for combination therapy with ICIs. The a2v‐mab activity targets pH regulation and elicits a weak ADCC response and can therefore mobilize innate immunity against tumor cells.

V‐ATPase has been shown to be abundantly expressed on hepatocellular cancer (HCC) cells and on tumor associated macrophages, making it a broadly and highly expressed pH regulator in the HCC microenvironment [60]. Results of the present study indicate that the V‐ATPase‐V0a2 isoform is expressed on malignant ovarian cells as well as infiltrating immune cells and is very highly expressed in the ovarian TME. This makes it a strong potential target with a two‐pronged approach to treat both malignant cells as well as tumor‐associated immune cells.

## 5 Conclusion

Targeting tumor‐associated vacuolar‐ATPase overcomes TME acidity‐driven immune dysfunction and can be an effective strategy for immune‐mediated tumor control in OVCA patients.

## Conflict of interest

The authors declare no conflict of interest.

## Author contributions

AK and GKK conceived, designed, and performed experiments, interpreted results, and undertook statistical analyses and manuscript writing. SAI participated in data organization and was involved in drafting the manuscript. VER, SS, MB, ANY, SL, and SF performed experiments. AGS helped with data interpretation and manuscript editing. JD provided clinical samples for the study. KDB was involved with the study design, data interpretation, and allocation of funds for the work and manuscript preparation.

## Supporting information


**Fig. S1.** V‐ATPase‐V0a2 expression on the tumor associated macrophages and neutrophils in human ovarian TME. (A) Immunohistochemical analysis of the serial sections from human OVCA tissues showing expression of macrophages (CD68 staining) and V‐ATPase‐V0a2 expression in the same tumor areas (*n* = 10). Representative images of two different OVCA tissues (i and ii) are shown here. Magnification 40X (scale bar‐50 µm). (B) Confocal microscopy analysis showing coexpression of V‐ATPAse‐V0a2 (red) on the tumor associated macrophages (CD68; green). Merged areas (in yellow) show coexpression of CD68 and V‐ATPase‐V0a2 in the ovarian TME; scale bar‐100 µm. The experiment was repeated three times (*n* = 3) (C) Immunohistochemical analysis of the serial sections from human OVCA tissues showing expression of neutrophils (using anti‐neutrophil‐ elastase staining) and V‐ATPase‐V0a2 expression in the same tumor areas (*n* = 10). Representative images of two different OVCA tissues (i and ii) are shown here at magnification 40X; scale bar‐50 µm. (D) Confocal microscopy analysis showing coexpression of V‐ATPAse‐V0a2 (red) on the tumor associated neutrophils (neutrophil elastase; green). Merged areas (in yellow) show coexpression of neutrophils and V‐ATPase‐v0a2 in the ovarian TME; scale bar‐50 µm. The experiment was repeated three times (*n* = 3).

**Fig. S2.** a2v‐mAb treatment resulted in no adverse effects in terms of gross weight in female athymic nude mice. The mean ± S.D body weight (g) of female athymic nude mice per group (*n* = 8) in a2v‐mAb‐treated and control (Mouse IgG) mice. The weight was measured from the day 0, which is the day of the first antibody injection, after palpable tumor appearance. The was no difference in the mean weight of the control and a2v‐mAb‐treated tumors as calculated by Student's *t*‐test (*P* = ns).

**Fig. S3.** Various strategies tested to determine the therapeutic efficacy of a2v‐mAb in OVCA: Anti‐V‐ATPase monoclonal antibody (a2v‐mAb) treatment schedule in OVCA (OVCA) xenograft model. (A) Intraperitoneal injection. Human OVCA cells (A2780) were implanted subcutaneously into the left upper flank of 3‐to‐4‐week‐old nude mice. Upon appearance of palpable tumors, intraperitoneal injections of a2v‐mAB (100 µg; three doses at 48 h interval) were given. Ms IgG control antibody was injected in another group of mice in the same way (*n* = 3/group). (B) Tumor site injection. Human OVCA cells (A2780) were implanted subcutaneously into the left upper flank of nude mice. Upon appearance of palpable tumors, a2v‐mAb injections (100 µg; 3 doses at 48h interval) were given at the site of tumor (*n* = 3/group). (B) Simultaneous injection of OVCA cells and a2v‐mAB. Human OVCA cells (A2780) were mixed with a2v‐mAb (100 µg) and injected into the left upper flank nude mice. Tumors were monitored for growth in a2v‐mAb vs Ms IgG control injected mice (*n* = 3/group).

**Fig. S4.** a2v‐mAb treatment significantly alters the immune cytokines and chemokines in tumors from treated nude mice. RNA sequencing analysis was performed using NGS using Mouse inflammation and immunity panel containing primers for 485 genes. The scatter plot compares the normalized expression of every gene on the array between the two selected groups by plotting them against one another to quickly visualize large gene expression changes. The central line indicates unchanged gene expression. The dotted lines indicate the selected fold regulation threshold. Genes showing more than twofold upregulation (*P* < 0.05) are highlighted as yellow dots. Genes showing more than twofold down regulated expression (*P* < 0.05) are marked in dark blue dots. *n* = 5 in control and *n* = 4 in a2v‐mAb tumor tissues. Statistical analysis was performed using Student's *t*‐test.

**Fig. S5.** *In vivo* a2v‐mAb treatment increases IFN‐gamma expression in ovarian tumors in nude mice. Intratumoral expression levels of IFNƳ mRNA. The total RNA was extracted from tumors, and the cytokine expression was determined by real‐time RT‐PCR. A higher expression of antitumor cytokine, IFN‐Ƴ was detected in tumors from a2v‐mAb‐treated mice compared to control (*P* = 0.0079). Data from *n* = 5 mice each in control and a2v‐mAb‐treated mice was analyzed and depicted as mean ± S.D. *P* < 0.05 was considered as statistically significant using Mann–Whitney test.

**Fig. S6.** a2v‐mAb antibody displays ADCC activity. ADCC is a mechanism for killing target cancer cells using IgG antibody‐based drugs that occurs by interaction of Fc portion of target‐bound antibodies to FcγRIIIa receptors on the cell surface of effector cells (T cells). *In vitro* treatment of target cells (B cells) with a2v‐mAb activated the NFAT pathway (expressing luciferase) in the effector T cells that causes target cell death. *X* axis represents a2v‐mAb log 10 concentrations. *Y* axis represents ADCC biological activity as determined by luciferase expressing N‐FAT activation in T cells. Anti‐CD20 antibody was used as positive IgG1 control. No antibody treatment was used as negative control. Mouse IgG1 control treatment did not elicit ADCC activity. The experiment was repeated thrice.

**Fig. S7.** a2v‐mAb treatment does not alter OVCA cell proliferation *in vitro*. Human OVCA cells (A2780) were treated with 300 µg of a2v‐mAb *in vitro* for 48 h at 37 °C, 5% CO_2_. The cell viability was determined by MTS colorimetric assay. Mouse IgG treatment was given in the control OVCA cells. The percent cell survival was calculated using no treatment group as 100% survival. (A) A2780 cells observed under light microscopy (10× and 20×; scale bar‐20 µm). Right panel: Ms IgG control treated A2780 cells. Left panel: A2780 cells treated with a2v‐mAb. (B) Percent cell survival in a2v‐mAb vs control cells depicted as mean ± SD of three values; statistical analysis performed using Student's *t*‐test.

## Data Availability

The raw data including RNA sequencing analysis data are available from the corresponding author upon reasonable request.
