# Supplementary figures and images for: In vivo anti‐V‐ATPase antibody treatment delays ovarian tumor growth by increasing antitumor immune responses
Source: Mol Oncol. 2020 Sep 10;14(10):2436–54. doi: 10.1002/1878-0261.12782 (PMC7530789; doi:10.1002/1878-0261.12782)

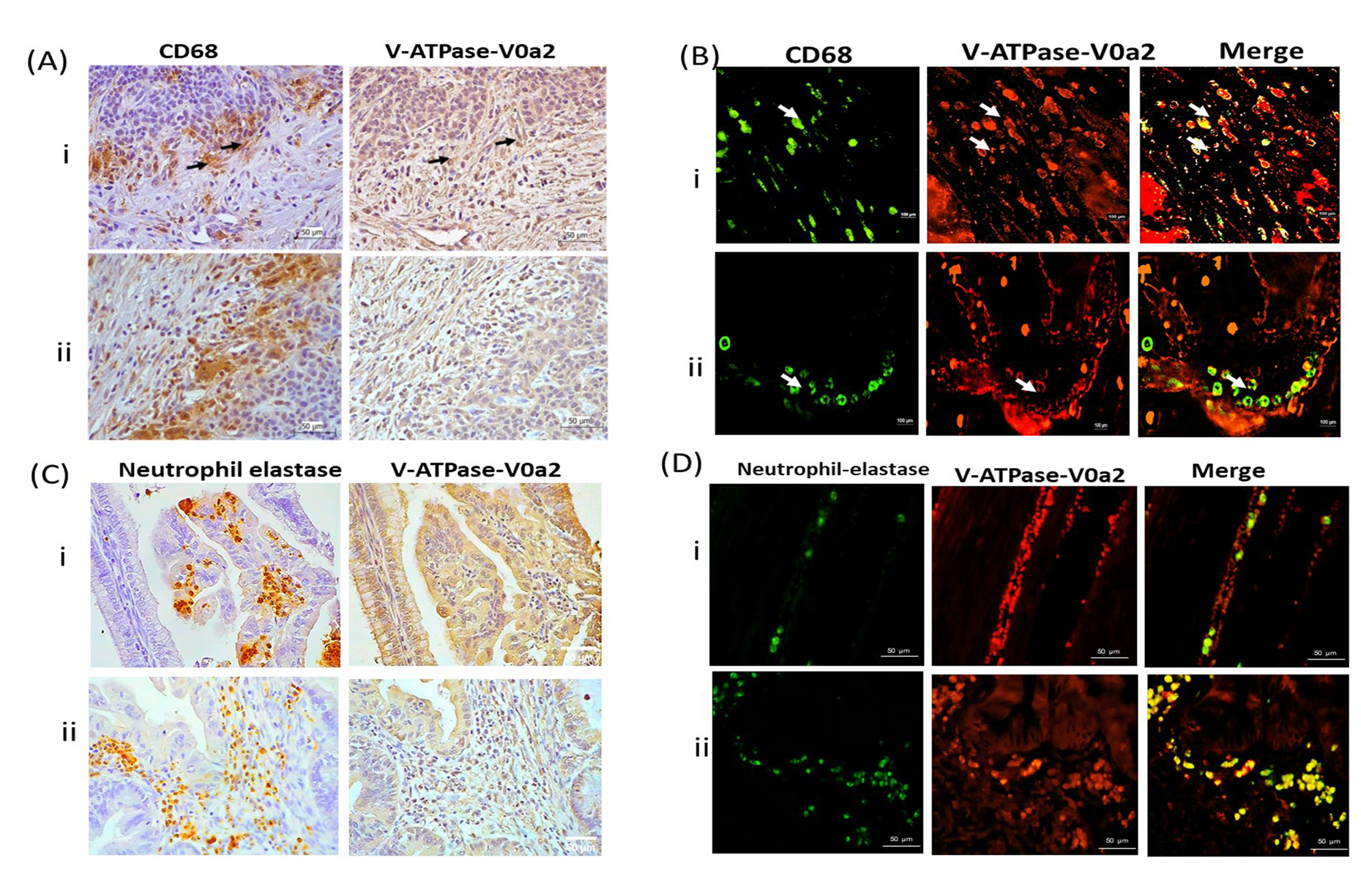

Supplement: Supplementary file 1 — Fig. S1. V‐ATPase‐V0a2 expression on the tumor associated macrophages and neutrophils in human ovarian TME. (A) Immunohistochemical analysis of the serial sections from human OVCA tissues showing expression of macrophages (CD68 staining) and V‐ATPase‐V0a2 expression in the same tumor areas (n = 10). Representative images of two different OVCA tissues (i and ii) are shown here. Magnification 40X (scale bar‐50 µm). (B) Confocal microscopy analysis showing coexpression of V‐ATPAse‐V0a2 (red) on the tumor associated macrophages (CD68; green). Merged areas (in yellow) show coexpression of CD68 and V‐ATPase‐V0a2 in the ovarian TME; scale bar‐100 µm. The experiment was repeated three times (n = 3) (C) Immunohistochemical analysis of the serial sections from human OVCA tissues showing expression of neutrophils (using anti‐neutrophil‐ elastase staining) and V‐ATPase‐V0a2 expression in the same tumor areas (n = 10). Representative images of two different OVCA tissues (i and ii) are shown here at magnification 40X; scale bar‐50 µm. (D) Confocal microscopy analysis showing coexpression of V‐ATPAse‐V0a2 (red) on the tumor associated neutrophils (neutrophil elastase; green). Merged areas (in yellow) show coexpression of neutrophils and V‐ATPase‐v0a2 in the ovarian TME; scale bar‐50 µm. The experiment was repeated three times (n = 3). [file MOL2-14-2436-s001.tif]

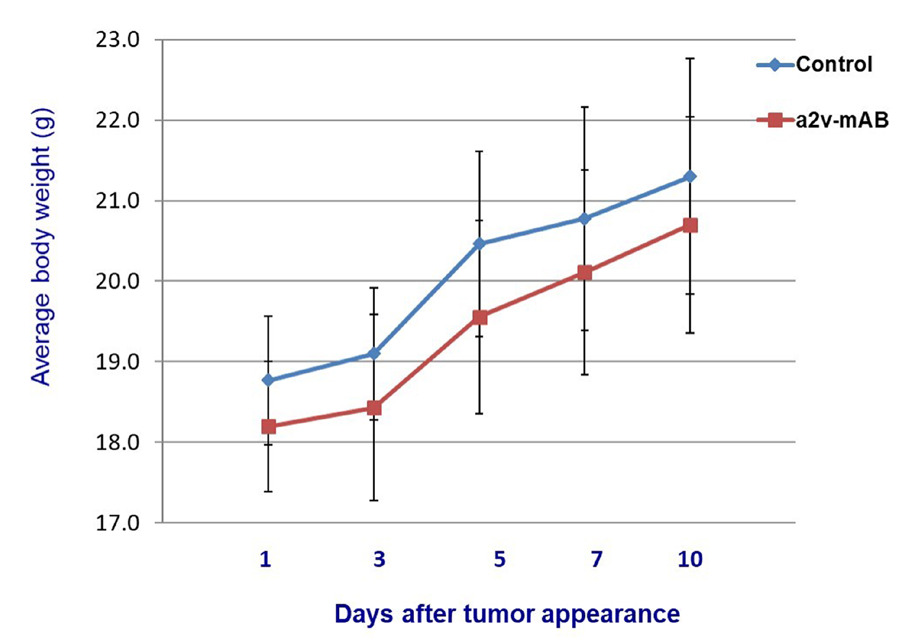

Supplement: Supplementary file 2 — Fig. S2. a2v‐mAb treatment resulted in no adverse effects in terms of gross weight in female athymic nude mice. The mean ± S.D body weight (g) of female athymic nude mice per group (n = 8) in a2v‐mAb‐treated and control (Mouse IgG) mice. The weight was measured from the day 0, which is the day of the first antibody injection, after palpable tumor appearance. The was no difference in the mean weight of the control and a2v‐mAb‐treated tumors as calculated by Student's t‐test (P = ns). [file MOL2-14-2436-s002.tif]

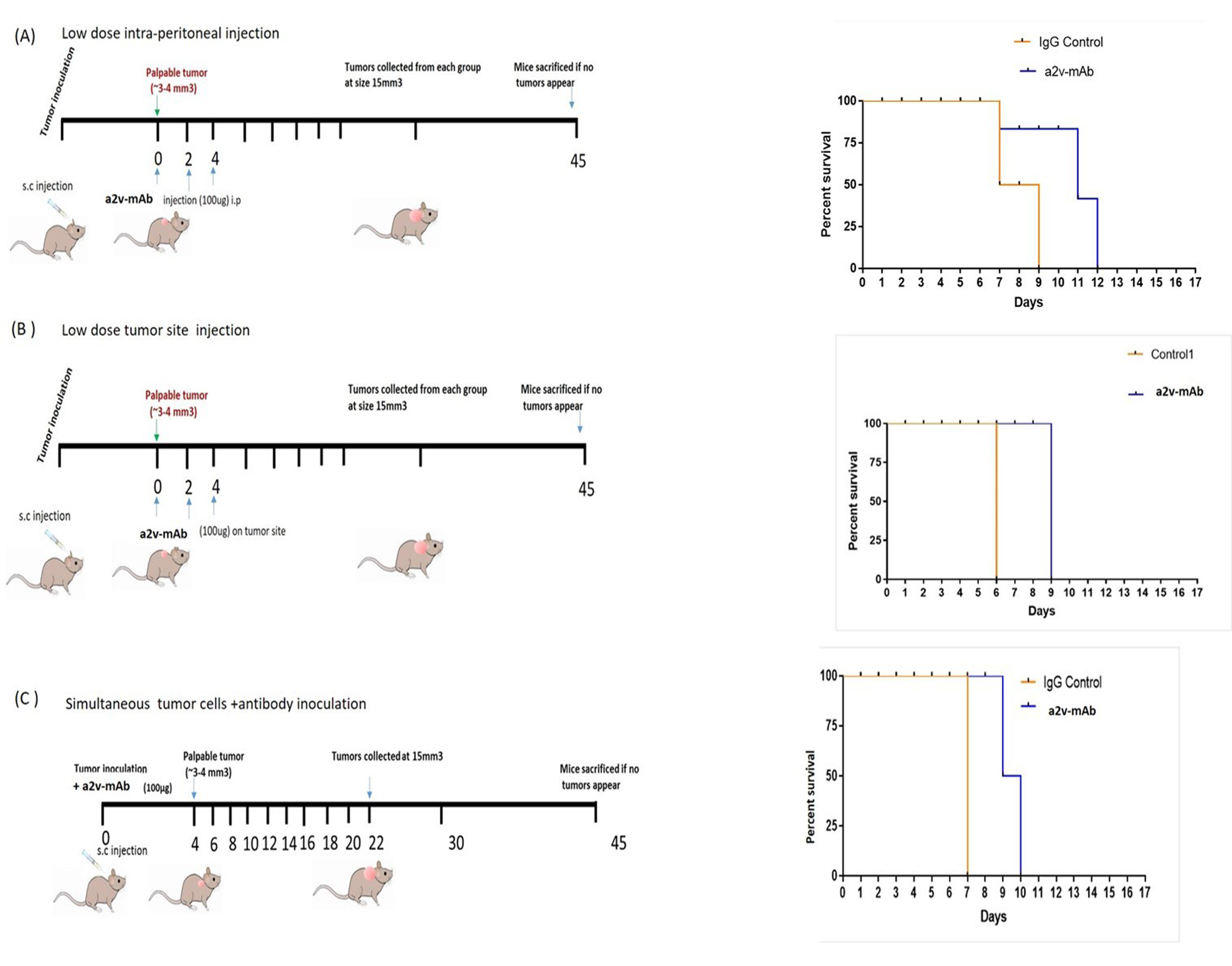

Supplement: Supplementary file 3 — Fig. S3. Various strategies tested to determine the therapeutic efficacy of a2v‐mAb in OVCA: Anti‐V‐ATPase monoclonal antibody (a2v‐mAb) treatment schedule in OVCA (OVCA) xenograft model. (A) Intraperitoneal injection. Human OVCA cells (A2780) were implanted subcutaneously into the left upper flank of 3‐to‐4‐week‐old nude mice. Upon appearance of palpable tumors, intraperitoneal injections of a2v‐mAB (100 µg; three doses at 48 h interval) were given. Ms IgG control antibody was injected in another group of mice in the same way (n = 3/group). (B) Tumor site injection. Human OVCA cells (A2780) were implanted subcutaneously into the left upper flank of nude mice. Upon appearance of palpable tumors, a2v‐mAb injections (100 µg; 3 doses at 48h interval) were given at the site of tumor (n = 3/group). (B) Simultaneous injection of OVCA cells and a2v‐mAB. Human OVCA cells (A2780) were mixed with a2v‐mAb (100 µg) and injected into the left upper flank nude mice. Tumors were monitored for growth in a2v‐mAb vs Ms IgG control injected mice (n = 3/group). [file MOL2-14-2436-s003.tif]

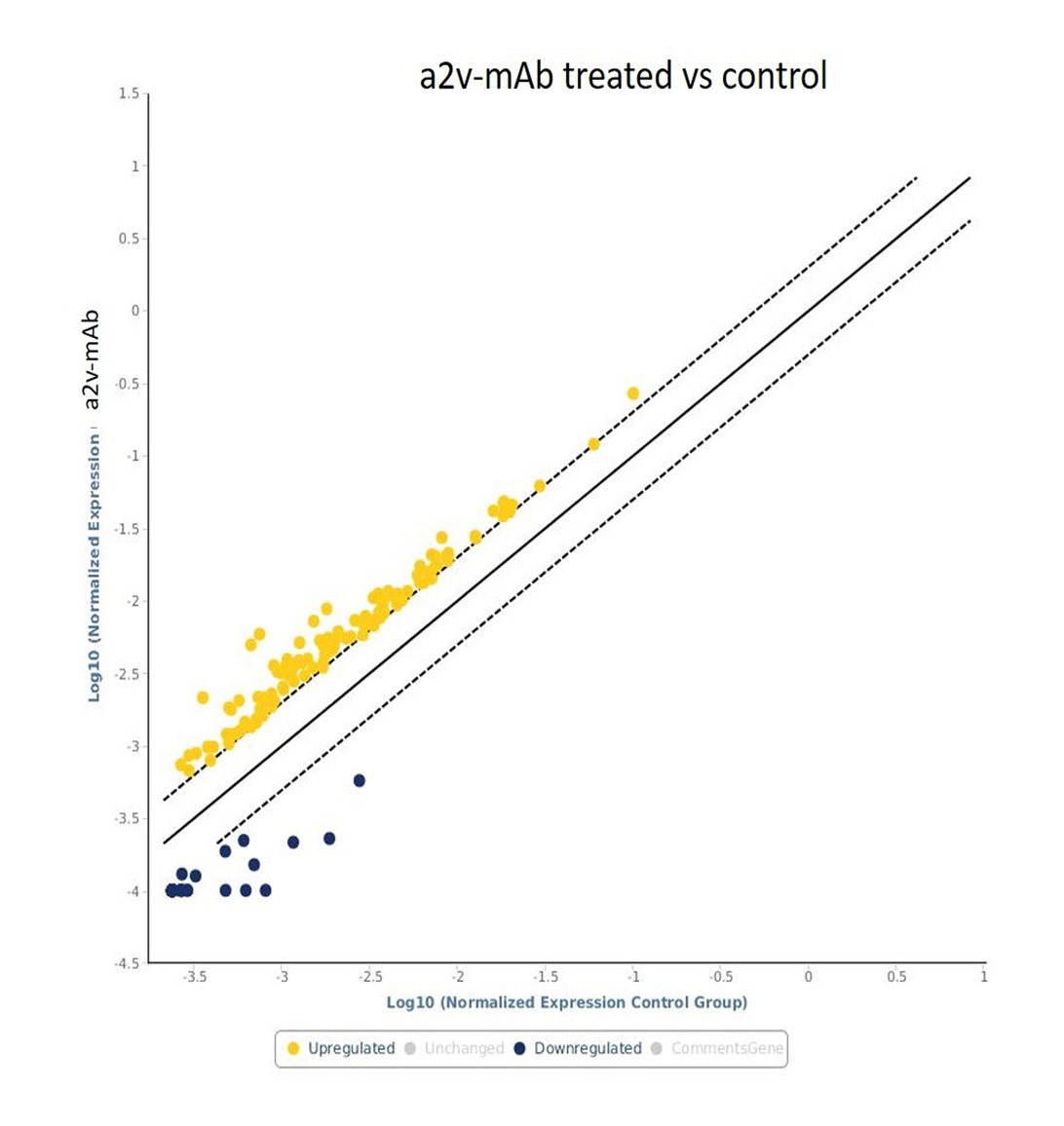

Supplement: Supplementary file 4 — Fig. S4. a2v‐mAb treatment significantly alters the immune cytokines and chemokines in tumors from treated nude mice. RNA sequencing analysis was performed using NGS using Mouse inflammation and immunity panel containing primers for 485 genes. The scatter plot compares the normalized expression of every gene on the array between the two selected groups by plotting them against one another to quickly visualize large gene expression changes. The central line indicates unchanged gene expression. The dotted lines indicate the selected fold regulation threshold. Genes showing more than twofold upregulation (P < 0.05) are highlighted as yellow dots. Genes showing more than twofold down regulated expression (P < 0.05) are marked in dark blue dots. n = 5 in control and n = 4 in a2v‐mAb tumor tissues. Statistical analysis was performed using Student's t‐test. [file MOL2-14-2436-s004.tif]

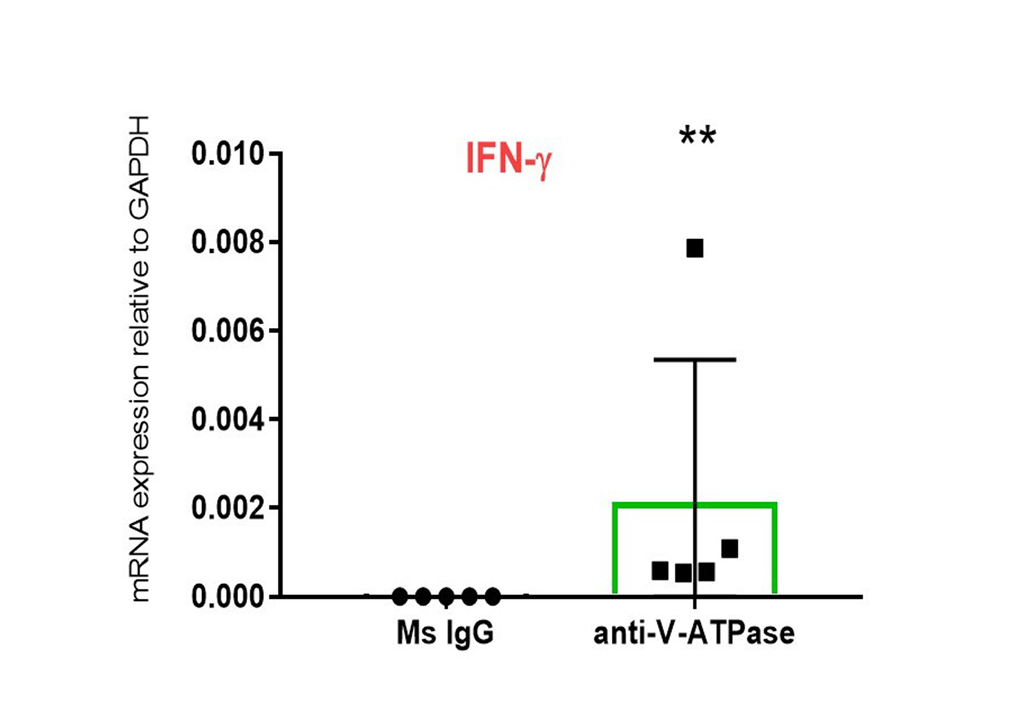

Supplement: Supplementary file 5 — Fig. S5. In vivo a2v‐mAb treatment increases IFN‐gamma expression in ovarian tumors in nude mice. Intratumoral expression levels of IFNƳ mRNA. The total RNA was extracted from tumors, and the cytokine expression was determined by real‐time RT‐PCR. A higher expression of antitumor cytokine, IFN‐Ƴ was detected in tumors from a2v‐mAb‐treated mice compared to control (P = 0.0079). Data from n = 5 mice each in control and a2v‐mAb‐treated mice was analyzed and depicted as mean ± S.D. P < 0.05 was considered as statistically significant using Mann–Whitney test. [file MOL2-14-2436-s005.tif]

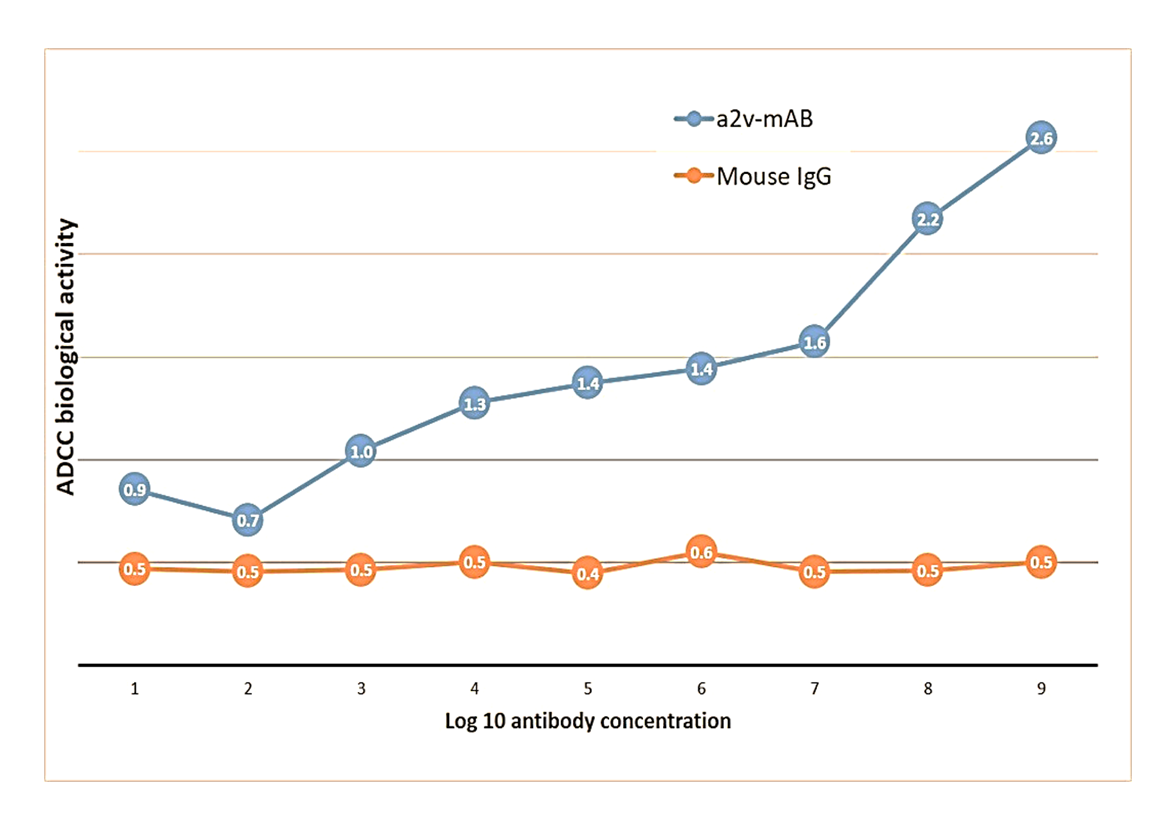

Supplement: Supplementary file 6 — Fig. S6. a2v‐mAb antibody displays ADCC activity. ADCC is a mechanism for killing target cancer cells using IgG antibody‐based drugs that occurs by interaction of Fc portion of target‐bound antibodies to FcγRIIIa receptors on the cell surface of effector cells (T cells). In vitro treatment of target cells (B cells) with a2v‐mAb activated the NFAT pathway (expressing luciferase) in the effector T cells that causes target cell death. X axis represents a2v‐mAb log 10 concentrations. Y axis represents ADCC biological activity as determined by luciferase expressing N‐FAT activation in T cells. Anti‐CD20 antibody was used as positive IgG1 control. No antibody treatment was used as negative control. Mouse IgG1 control treatment did not elicit ADCC activity. The experiment was repeated thrice. [file MOL2-14-2436-s006.tif]

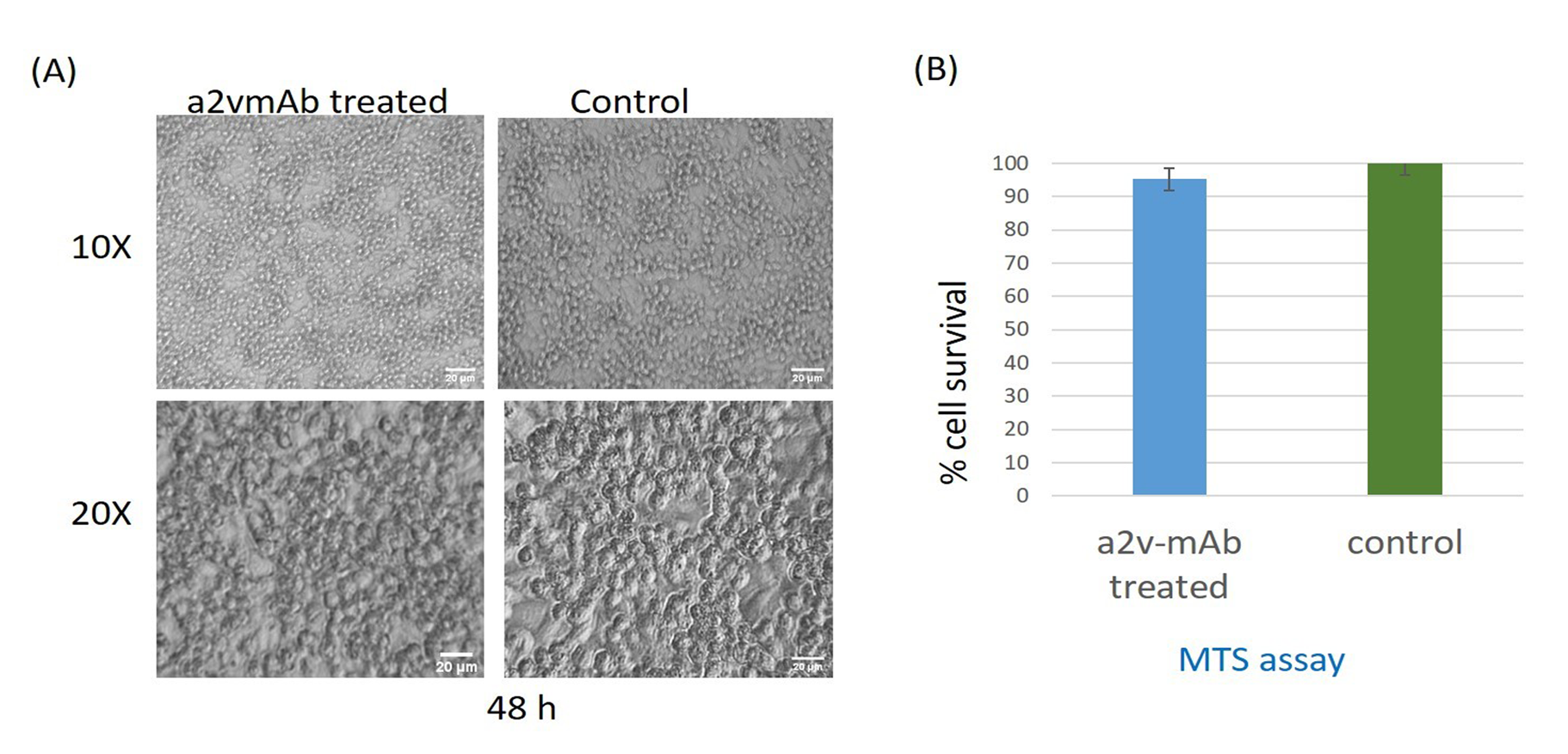

Supplement: Supplementary file 7 — Fig. S7. a2v‐mAb treatment does not alter OVCA cell proliferation in vitro. Human OVCA cells (A2780) were treated with 300 µg of a2v‐mAb in vitro for 48 h at 37 °C, 5% CO2. The cell viability was determined by MTS colorimetric assay. Mouse IgG treatment was given in the control OVCA cells. The percent cell survival was calculated using no treatment group as 100% survival. (A) A2780 cells observed under light microscopy (10× and 20×; scale bar‐20 µm). Right panel: Ms IgG control treated A2780 cells. Left panel: A2780 cells treated with a2v‐mAb. (B) Percent cell survival in a2v‐mAb vs control cells depicted as mean ± SD of three values; statistical analysis performed using Student's t‐test. [file MOL2-14-2436-s007.tif]
